# High content 3D imaging by dual-view oblique plane microscopy

**DOI:** 10.1093/pnasnexus/pgaf370

**Published:** 2025-11-26

**Authors:** Hugh Sparks, Leo Rowe-Brown, Yuriy Alexandrov, Nils Gustafsson, Liuba Dvinskikh, Nathan Curry, Jayne Culley, Martin Lee, Alix Le Marois, Colin D H Ratcliffe, Thomas A Phillips, Claudia Owczarek, Mar Arias Garcia, Montserrat Llanses, Theresa Suckert, Joffrey Pelletier, Carme Cortina, Wenzhi Hong, Edwin Garcia, Zhizhen Xu, Shengjie Zhang, Giorgio Stassi, Eduard Batlle, Julien Colombelli, Maddy Parsons, Chris Bakal, Neil O Carragher, Erik Sahai, Chris Dunsby

**Affiliations:** Light Community, Department of Physics, Imperial College London, South Kensington Campus, London SW7 2AZ, United Kingdom; Tumour Cell Biology Laboratory, The Francis Crick Institute, 1 Midland Road, London NW1 1AT, United Kingdom; Light Community, Department of Physics, Imperial College London, South Kensington Campus, London SW7 2AZ, United Kingdom; Tumour Cell Biology Laboratory, The Francis Crick Institute, 1 Midland Road, London NW1 1AT, United Kingdom; Light Community, Department of Physics, Imperial College London, South Kensington Campus, London SW7 2AZ, United Kingdom; Tumour Cell Biology Laboratory, The Francis Crick Institute, 1 Midland Road, London NW1 1AT, United Kingdom; Light Community, Department of Physics, Imperial College London, South Kensington Campus, London SW7 2AZ, United Kingdom; Tumour Cell Biology Laboratory, The Francis Crick Institute, 1 Midland Road, London NW1 1AT, United Kingdom; Light Community, Department of Physics, Imperial College London, South Kensington Campus, London SW7 2AZ, United Kingdom; Tumour Cell Biology Laboratory, The Francis Crick Institute, 1 Midland Road, London NW1 1AT, United Kingdom; Light Community, Department of Physics, Imperial College London, South Kensington Campus, London SW7 2AZ, United Kingdom; Tumour Cell Biology Laboratory, The Francis Crick Institute, 1 Midland Road, London NW1 1AT, United Kingdom; Cancer Research UK Scotland Centre (Edinburgh), Institute of Genetics and Cancer, University of Edinburgh, Western General Hospital, Crewe Road South, Edinburgh EH4 2XU, United Kingdom; Cancer Research UK Scotland Centre (Edinburgh), Institute of Genetics and Cancer, University of Edinburgh, Western General Hospital, Crewe Road South, Edinburgh EH4 2XU, United Kingdom; Tumour Cell Biology Laboratory, The Francis Crick Institute, 1 Midland Road, London NW1 1AT, United Kingdom; Tumour Cell Biology Laboratory, The Francis Crick Institute, 1 Midland Road, London NW1 1AT, United Kingdom; Randall Centre for Cell and Molecular Biophysics, King's College London, Guy's Campus, London SE1 1UL, United Kingdom; Randall Centre for Cell and Molecular Biophysics, King's College London, Guy's Campus, London SE1 1UL, United Kingdom; Dynamical Cell Systems Team, Division of Cancer Biology, The Institute of Cancer Research-Chester Beatty Laboratories, 237 Fulham Road, London SW3 6JB, United Kingdom; Institute for Research in Biomedicine (IRB Barcelona), Barcelona Institute of Science and Technology (BIST), c. Baldiri Reixac 10, 08028 Barcelona, Spain; Institute for Research in Biomedicine (IRB Barcelona), Barcelona Institute of Science and Technology (BIST), c. Baldiri Reixac 10, 08028 Barcelona, Spain; Laboratory of Cancer Metabolism, ONCOBELL Program, Bellvitge Biomedical Research Institute (IDIBELL), 08908 L'Hospitalet de Llpbregat, Barcelona, Spain; Department of Physiological Sciences, Faculty of Medicine and Health Sciences, University of Barcelona, 08908 L'Hospitalet de Llobregat, Barcelona, Spain; Institute for Research in Biomedicine (IRB Barcelona), Barcelona Institute of Science and Technology (BIST), c. Baldiri Reixac 10, 08028 Barcelona, Spain; Centro de Investigación Biomédica en Red de Cáncer (CIBERONC), Barcelona, Spain; Light Community, Department of Physics, Imperial College London, South Kensington Campus, London SW7 2AZ, United Kingdom; Light Community, Department of Physics, Imperial College London, South Kensington Campus, London SW7 2AZ, United Kingdom; Tumour Cell Biology Laboratory, The Francis Crick Institute, 1 Midland Road, London NW1 1AT, United Kingdom; Light Community, Department of Physics, Imperial College London, South Kensington Campus, London SW7 2AZ, United Kingdom; Light Community, Department of Physics, Imperial College London, South Kensington Campus, London SW7 2AZ, United Kingdom; Department of Precision Medicine in Medical, Surgical and Critical Care (Me.Pre.C.C), University of Palermo, Palermo 90127, Italy; IRCCS SYNLAB SDN, Naples 80143, Italy; Institute for Research in Biomedicine (IRB Barcelona), Barcelona Institute of Science and Technology (BIST), c. Baldiri Reixac 10, 08028 Barcelona, Spain; Centro de Investigación Biomédica en Red de Cáncer (CIBERONC), Barcelona, Spain; Institució Catalana de Recerca i Estudis Avançats (ICREA), 08010 Barcelona, Spain; Institute for Research in Biomedicine (IRB Barcelona), Barcelona Institute of Science and Technology (BIST), c. Baldiri Reixac 10, 08028 Barcelona, Spain; Randall Centre for Cell and Molecular Biophysics, King's College London, Guy's Campus, London SE1 1UL, United Kingdom; Dynamical Cell Systems Team, Division of Cancer Biology, The Institute of Cancer Research-Chester Beatty Laboratories, 237 Fulham Road, London SW3 6JB, United Kingdom; Cancer Research UK Scotland Centre (Edinburgh), Institute of Genetics and Cancer, University of Edinburgh, Western General Hospital, Crewe Road South, Edinburgh EH4 2XU, United Kingdom; Tumour Cell Biology Laboratory, The Francis Crick Institute, 1 Midland Road, London NW1 1AT, United Kingdom; Light Community, Department of Physics, Imperial College London, South Kensington Campus, London SW7 2AZ, United Kingdom; Tumour Cell Biology Laboratory, The Francis Crick Institute, 1 Midland Road, London NW1 1AT, United Kingdom

## Abstract

Oblique plane microscopy (OPM) is a form of light-sheet fluorescence microscopy (LSFM) employing a single microscope objective at the sample for both fluorescence excitation and detection. Dual-view OPM (dOPM) is an optically folded form of OPM. We present an improved dOPM system employing a 60×/1.2NA water immersion primary objective and measure the spatial resolution and fluorescence collection efficiency for illumination angles of 35° and 45° with respect to the coverslip. Illumination at 35° provides slightly better lateral resolution and collection efficiency. Collection efficiency measurements are compared to a full vectorial raytracing simulation of the system. Using a light-sheet angle of 35°, the median bead FWHM for 100 nm diameter fluorescent beads in x, y, and z and the optical sectioning strength were measured over a volume of 100 × 100 × 100 μm^3^ to be 0.29, 0.31, 0.83, and 2.45–3.00 μm, respectively when the two dOPM views are fused. We demonstrate less photobleaching in time-lapse dOPM of live mEmerald-expressing organoids compared to widefield epi-fluorescence z-stack imaging under the condition of equal detected fluorescence signal from a point object in focus. We demonstrate dOPM for multifield-of-view 3D imaging of biological samples in 96-well plates and apply it to imaging cells in collagen gel and quantifying the FUCCI cell-cycle reporter to provide drug dose–response curves in spheroids. We also use it to perform time-lapse multifield-of-view imaging and demonstrate the detection of organoid lumen closure and reopening, organoid migration within a collagen gel and observing dynamic events in arrays of ex vivo tissue slices.

Significance Statement Dual-view oblique plane microscopy is an optically folded oblique plane microscopy technique enabling multiview light-sheet imaging. The use of a polarizing beam splitter cube reduces the fluorescence collection efficiency but allows a single high-NA lens to function as both the secondary and tertiary objective, achieving a relatively high overall NA in a relatively compact lower-cost system. We demonstrate similar spatial resolution to previous reports using low-working distance high NA tertiary objectives and that this is maintained over a ∼5-fold greater axial range for fluorescent spheres in a homogenous medium. We present detailed measurements of optical collection efficiency, calculations of light dose and measurements of photobleaching for dOPM. dOPM is demonstrated for a wide range of biomedical imaging applications.

## Introduction

Light-sheet fluorescence microscopy (LSFM) provides fast 3D imaging with low light dose to biological samples compared to confocal microscopy (1). Conventional LSFM employs separate objective lenses for sample illumination and fluorescence detection, which places constraints on how the sample can be mounted (2). In order to use LSFM to image arrays of samples in multiwell plates, it is possible to use open-top LSFM configurations that avoid or compensate for the aberrations encountered when illuminating and imaging through a tilted coverslip (3–6). Alternatively, oblique plane microscopy (OPM) allows illumination and fluorescence imaging of a tilted plane using a single objective lens (7, 8). OPM builds on the remote-refocusing approach of Botcherby et al. (9, 10) and uses a set of three microscopes in series with the optical axis of the third microscope at an angle to the first two. Remote refocusing by axial motion of the secondary objective can be used to achieve high-speed 3D imaging (11). Rapid lateral scanning can be achieved through the use of a galvo in a plane conjugate to the pupil of the primary objective (12), and the effective numerical aperture (NA) of OPM can be improved by using a high NA tertiary microscope objective with small working distance (13–15).

However, the spatial resolution of LSFM is generally anisotropic and poorer in the axial direction. In open-top LSFM, the axial resolution can be improved by employing axially swept illumination and detection at the expense of additional light dose to the specimen (16) or by using lattice light-sheet illumination (17). The implementation of line illumination and confocal slit detection can reduce scattered light from out-of-focus planes at the expense of higher peak intensity (18). The spatial resolution can be made more isotropic in conventional LSFM by acquiring image data at multiple view angles and then fusing or deconvolving the data in post processing, and this has the additional advantage of reducing light-sheet shadowing and streak artifacts (19, 20). We demonstrated dual-view OPM imaging using an optically folded dual-mirror approach termed dual-view OPM (dOPM) (21), which enabled two views 90° apart to be obtained. An alternative approach using galvo mirrors to switch between two views (22) does not require the use of a polarizing beam-splitter (PBS) in the detection path but employs an optical system with additional pupil relays.

In this article, we perform a detailed characterization of a dOPM system employing a 60×/1.2 NA water immersion primary objective where the calculated latitudinal and longitudinal NAs (of 1.2 and 1.03 for an OPM angle of 35°) are improved compared to our original work (40×/1.15 NA water lens with dOPM NAs of 0.93 and 0.58 (21)). We explore the trade-off between light-sheet illumination at 35° and 45° with respect to the coverslip and present measurements of spatial resolution and optical sectioning strength over a field of view (FOV) of 100 × 100 × 100 μm^3^. Using widefield epi-fluorescence imaging, we measure the collection efficiency of the dOPM system relative to that of the primary microscope alone. We also develop a methodology for determining the photobleaching of dOPM relative to widefield epi-fluorescence imaging with the primary microscope for equal fluorescence signals in the two systems for a point object in focus. This methodology is applied in live mEmerald-expressing organoids under time-lapse 3D imaging. Imaging using e.g. a spinning disk confocal system would cause a light dose that is no smaller than the widefield imaging light dose, and so this measurement provides an upper limit on the photobleaching due to dOPM compared to spinning-disk confocal imaging. Using measurements of the light-sheet thickness, we calculate that the light dose due to dOPM for our standard dOPM volume acquisition parameters is ∼10 times lower than widefield imaging. We demonstrate the application of dOPM to multi-FOV 3D imaging in 96-well plates for single cells embedded within a collagen gel and for single-cell readout of the FUCCI cell-cycle reporter for determining drug dose–response curves in a glioblastoma stem cell spheroid model. In addition, time-lapse multi-FOV 3D dOPM is used to image patient-derived triple-negative breast cancer organoids and to study cancer cell migration and division in ex vivo mouse lung tissue slices. These results demonstrate the strengths of the dOPM system—which includes the benefits of dual-view imaging—for performing multi-FOV and/or time-lapse imaging across multiple model biological systems.

## Results

### Spatial resolution

To assess the spatial resolution of the dOPM system, we imaged 100 nm diameter fluorescent beads embedded in 3D in agarose for the system configured at 35° and 45° OPM angles. The minimum OPM angle of the system is limited by the NA of O1 to 26° degrees, but this does not leave any NA free for generating a thin light sheet. Therefore, the first OPM angle of 35° was chosen as a compromise between maximizing the angle between the two views and maximizing the NA for fluorescence collection. The second angle of 45° was chosen as this represents the case where the two illumination light sheets are orthogonal and therefore where there is likely to be the greatest complementarity between the two views. Latitudinal and longitudinal NAs and the angle between the two views are given in Table S1 for a range of OPM angles.

**Fig. 1.**
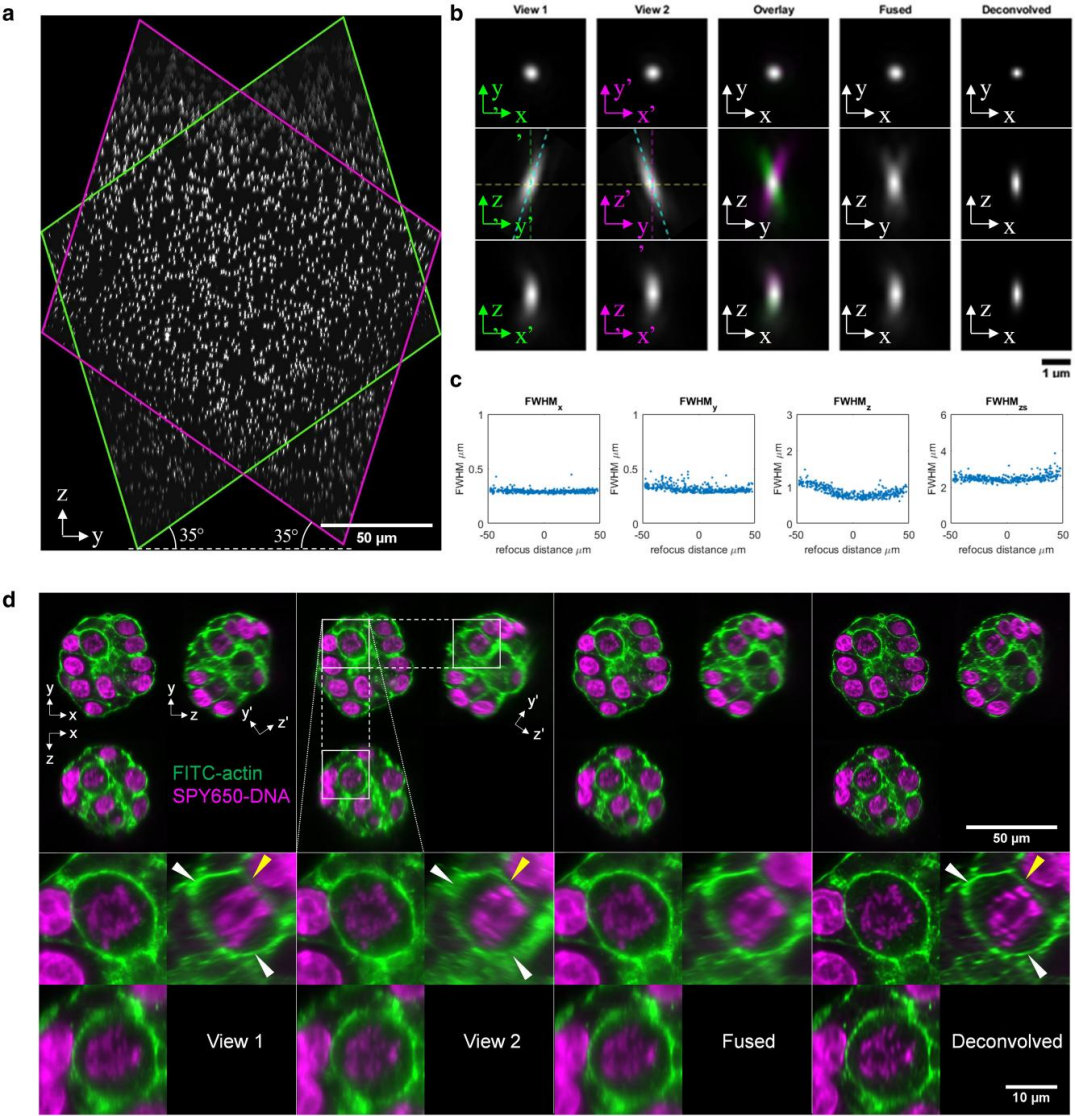
dOPM imaging of 100 nm fluorescent beads embedded in 3D in agarose and of fixed TNBC PDXOs in Cultrex. a–c) 100 nm fluorescent bead data obtained for a 35° OPM angle. a) MIP of fused bead volume along x direction. b) Orthoplanes through a representative bead for view 1, view 2, the fusion of views 1 and 2 and deconvolution of views 1 and 2. Images for view 1 and view 2 are shown in the frame of that view. c) Plots of fused bead image FWHM in the x, y, and z directions for all beads within a 100^3^ μm^3^ volume as a function of z position within volume. The right hand plot shows the results for the z sectioning. d) 3D dOPM imaging of GCRC1915 TNBC organoids. From left to right: view 1, view 2, fused, and deconvolved orthoplanes. Top row, full FOV; bottom row, zoomed region as indicated in top row. Green (actin), FITC-Phalloidin; magenta (nuclei), SPY650-DNA.

Maximum intensity projections (MIPs) of the resulting volume along the × direction are shown in Fig. 1 for the 35° OPM, together with orthogonal planes through the center of the volume (orthoplanes) of one exemplar bead. The full width at half maximum (FWHM) of bead images from the fused data meeting the inclusion criteria detailed in the Methods within a 100^3^ μm^3^ volume are shown in Fig. 1c as a function of their position in *z* relative to the zero-defocus plane *z* = 0, showing the consistency of the spatial resolution over the image volume. The corresponding images for the 45° OPM angle are shown in Fig. S1. Median bead FWHM and corresponding interquartile ranges (IQRs) for the fused data are reported in Table 1 and graphically in Fig. S2. Data for individual views, fused data, and deconvolved data for both OPM angles are shown in Figs. S3 and S4. The FWHM in the y-direction is smaller for an OPM angle of 35° compared to 45°, as expected from the calculated theoretical longitudinal NA (see Methods) shown in Table S1. The axial FWHM is slightly smaller for an OPM angle of 45°, which we attribute to the greater tilt of the PSF with respect to the optical axis. The z-sectioning results are similar for both OPM angles. Consistent with results from our previous publication (21), we do not see resolution improvement on fusing view 1 and view 2, see Figs. S3 and S4. Deconvolution can lead to resolution improvement, but as this process depends on exactly how the deconvolution is performed and requires additional computational cost, and given the focus of this article is on high content imaging, the vast majority of the data presented in this article is therefore fused rather than deconvolved.

**Table 1.** Median and IQR bead image FWHM for two OPM angles (35° and 45°) and the percentage change for an angle of 45° relative to 35° for the fused data.

θ_OPM_ (°)	Median FWHM x ± IQR [CI]	Median FWHM y ± IQR [CI]	Median FWHM z ± IQR [CI]	Median FWHM z sectioning from bead ± IQR [CI]	Median FWHM z sectioning from thin sheet ± IQR [CI]
35	0.293 ± 0.019 [0.291, 0.295]	0.309 ± 0.035 [0.306, 0.313]	0.827 ± 0.211 [0.813, 0.845]	2.45 ± 0.181 [2.432, 2.462]	3.007 ± 0.135 [3.006, 3.008]
45	0.301 ± 0.022 [0.299, 0.302]	0.337 ± 0.037 [0.334, 0.341]	0.754 ± 0.238 [0.739, 0.765]	2.42 ± 0.217 [2.408, 2.451]	3.046 ± 0.226 [3.045, 3.048]
% change relative to 35	2.7	9.1	−8.8	−1.1	1.9

The 95% CI on the median is given in square brackets. Values are shown for a 100^3^ μm^3^ volume. The IQR values for the bead data indicate the spread in values over the whole imaged volume (xyz). The IQR values for the sectioning measured using a thin sheet describe the spread in values over the lateral (xy) extent of the FOV.

We also measured the z sectioning of the system by acquiring an image volume from a thin fluorescent sheet, see Methods, and the median and IQR are reported in Table 1 and Fig. S2b. The results show a higher FWHM compared to the z-sectioning data calculated from the fluorescent bead measurements. The z-sectioning obtained from the laterally integrated fluorescent bead signal will tend to provide a smaller value for the z-sectioning due to the difficulty of measuring the energy from the bead data at larger axial distances from the bead where the bead's energy is spread over a larger area on the camera. The data from the thin sheet are in line with the theoretical prediction of the light sheet FWHM of 2.8 μm based on the 6 mm diameter of the iris diaphragm (see system diagram in Fig. S5).

### Collection efficiency

Using the same approach as in our previous characterization of OPM (23), we chose to measure the collection efficiency of the dOPM system relative to that of a widefield epi-fluorescence microscope with identical primary objective (O1), tube lens (TL1), and camera. To do this, we imaged the same spatially isolated bead positioned in the center of the front focal plane of O1 with both widefield and dOPM collection optics, see Methods and Fig. S6. These results could then be compared to simulation results calculated using a simpler geometrical model of the dOPM collection efficiency and a full vectorial ray-tracing calculation, again see Methods.

The collection efficiency measurements are shown in Fig. S7 together with results from simulation. Figure S7a–c shows results from simulations assuming fluorophores tumbling rapidly compared to the fluorescence lifetime (hereafter rapidly tumbling fluorophores) and comparable experimental measurements performed with unpolarized excitation light. Figure S7d–f shows simulations performed assuming a population of randomly oriented static fluorophores excited by linearly polarized light with the electric field vector in the plane of the light sheet (hereafter static fluorophores) and comparable experimental measurements performed using linearly polarized excitation aligned with the transmission axis of the PBS. Figure S7 (a and b) and (d and e) show the simulated fluorescence intensity in the pupil of O3 using vectorial ray tracing. From left to right, Fig. S7c and f shows for three OPM angles: simulation results from the geometric model that assumes a perfect M3/7 mirror, the vectorial simulation results assuming a perfect mirror, a SiO_2_-coated silver mirror and a SiO-coated mirror, experimental results for an OPM angle of 0° performed using a silver protected mirror from Thorlabs (PF05-03-P01), and experimental results for OPM angles of 35° and 45° performed using a silver protected mirror from Edmund Optics (part number 89623). As described in the Methods, the numerical calculations (geometric and vectorial) include the transmission of O2/3 stated by the manufacturer and the experimentally measured transmission of collimated linearly polarized on-axis light double-passing through the PBS and QWP4.

Figure S7c and f shows that for the case of an assumed perfect M3/7 mirror, the much simpler geometric model provides results that are reasonably similar to the more detailed vectorial calculation (left-hand two conditions). The vectorial calculation however is needed to model the full effect of the reflection at M3/7, which results in lower calculated collection efficiencies (columns 3 and 4).

The experimental collection efficiency measurements are slightly below the vectorial simulations using ideal SiO_2_ or SiO-coated aluminum mirrors, and we note that the exact coating specifications of the Edmund Optics M3/7 mirrors used for nonzero OPM angles are not provided by the manufacturer. Overall, the experimentally measured relative dOPM collection efficiency for an OPM angle of 35° was 0.23 for polarized excitation (equivalent to the case of static fluorophores) and 0.19 for unpolarized excitation (equivalent to the case of rapidly tumbling fluorophores).

### dOPM photobleaching compared to widefield imaging for equal fluorescence signal

We assessed the photobleaching of dOPM imaging against nonsectioning widefield epi-fluorescence imaging in mEmerald-expressing organoids with the LED and laser illumination powers set so that equal fluorescence signal was collected by both methods from an in-focus bead in the center of the FOV, see Methods section and Fig. S8. Any spinning-disk confocal system will contain more optical elements in the collection path and therefore cannot have a higher collection efficiency than a widefield system. Thus, this measurement provides a comparison of dOPM against the best possible performance of a spinning-disk system.

MIPs of the resulting data are shown in Fig. S9a and b together with a graph of the change in signal over time in Fig. S9c. The average percentage drop in dOPM signal over the experiment was 11 ± 11% (median ± IQR) while the corresponding change for widefield imaging was 66 ± 10%.

To put these results into context, we also calculated the total light exposure for equivalent widefield and dOPM imaging acquisitions, see Fig. S9d. As with the experiments, this calculation was performed under the condition that the same fluorescence signal is obtained for a sample consisting of a point object placed in the center of the volume imaged. In Fig. S9d, the light dose of a single widefield image is defined to be 1 arbitrary unit (a.u.) for each plane in the image stack, resulting in a total dose of 151 widefield exposures to the sample; the total light dose to the sample is represented by the area of the blue-filled region. Using the worst case (widest) measurement of the light sheet, which was 3.0 μm obtained from imaging the thin fluorescent sheet (Table 1), and the measured dOPM relative collection efficiency (RC) for static fluorophores of 0.23 (see Methods, Collection efficiency), we also plot the light dose to the sample for dual-view dOPM imaging (red). Due to the lower light collection efficiency of dOPM compared to widefield imaging, the point object is exposed to a higher dose when the light sheet is centered on the object but is not exposed when acquiring image planes away from the object. The light dose to the sample due to either view alone is shown in yellow. Overall, the total light dose to the sample is a factor of 10.9 lower for dOPM compared to widefield imaging for imaging static fluorophores. The equivalent value is 9.0 for rapidly tumbling fluorophores.

### Imaging fixed triple-negative breast-cancer PDXOs

We next sought to apply dOPM to state-of-the-art models of human triple-negative breast cancer (TNBC) that have been generated by culturing patient-derived xenograft organoids (PDXOs) in 3D (24, 25). We imaged fixed TNBC organoids (GCRC1915) grown on a layer of Cultrex matrix using two spectral channels reporting F-actin (FITC-Phalloidin) and DNA (SPY650-DNA), see Fig. 1d. While both views captured the entire organoid volume, we noted differences where one view complemented the other; white arrowheads indicate F-actin staining present in view 1 not clearly seen in view 2, and the yellow arrowhead indicates staining present in view 2 not clearly seen in view 1. In the fused, and in particular in the deconvolved (see Methods, Image deskewing and registration) image shown in the bottom ZY view of Fig. 1d, cortical actin delineating the perimeter of a cell within the organoid is visualized more completely than in view 1 or view 2 alone.

### Live patient-derived triple-negative breast-cancer organoid 3D imaging

**Fig. 2.**
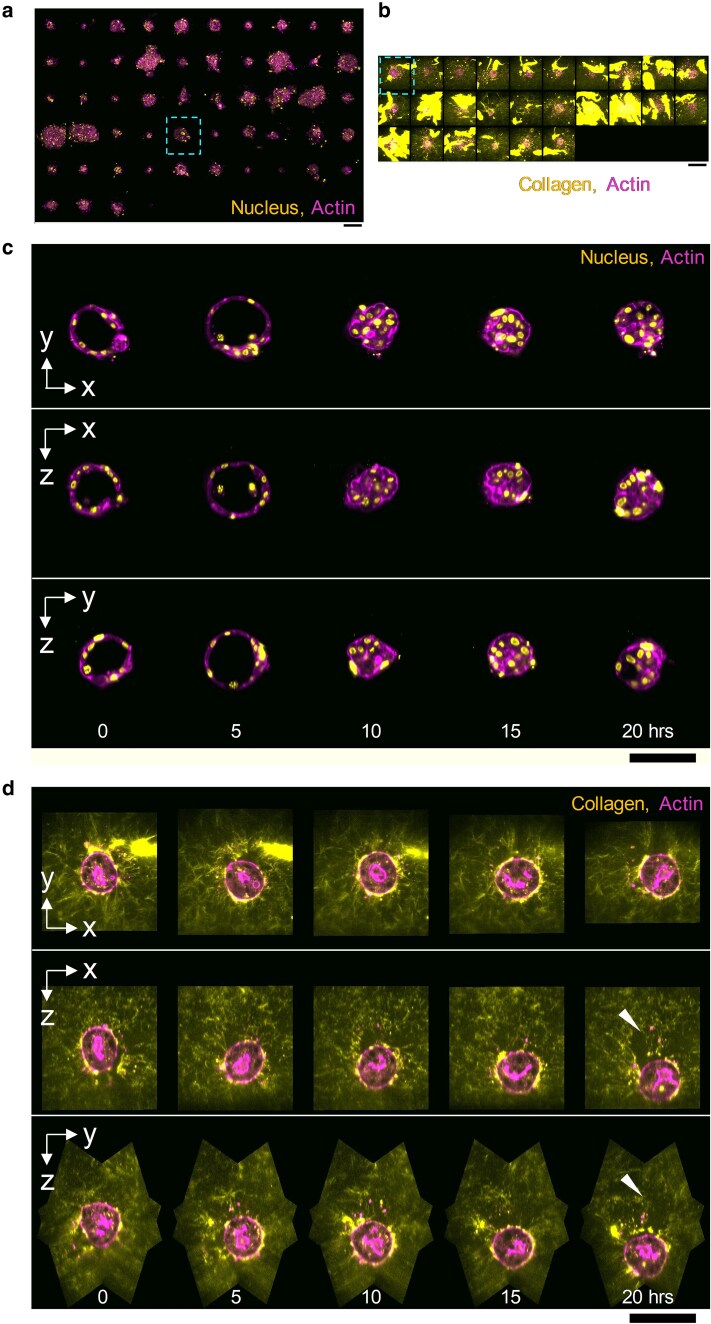
3D time-lapse multi-FOV imaging of primary TNBC organoids. 80 FOVs, with 48 time points acquired at 30 min intervals. Arrays of MIPs showing the FOVs imaged: a) organoids where nucleus and actin are labeled, b) organoids where collagen and actin are labeled. c) Orthoplanes from an organoid indicated by dashed box in (a) showing lumen closure and then reopening over time. d) Organoid indicated by dashed box in (b) showing migration of organoid through the collagen matrix over time. Collagen signal brightness has been adjusted to better show dimmer regions, which has caused saturation in brighter regions. Scale bars 100 m.

dOPM was used to image arrays of live primary TNBC organoids embedded in collagen and stained with exogenous labels for nucleus and actin, see Fig. 2a, and collagen and actin, see Fig. 2b. See also Movie S1. Figure 2c and Movie S2 show an example of an organoid where the lumen closes and then reforms. Figure 2d shows migration of the organoid by ∼46 µm (3D displacement) over 20 h, with shifts of ∼22 µm in x, 2 µm in y, and 41 µm in z directions. The residual hole in the collagen matrix is resolved in the xz and yz views, see white arrowheads and Movie S3. Movie S3 also reveals the organoid exerting changing forces on the collagen matrix as it migrates.

### Time-lapse 3D imaging of ex vivo tumor xenografts in precision-cut lung slices

**Fig. 3.**
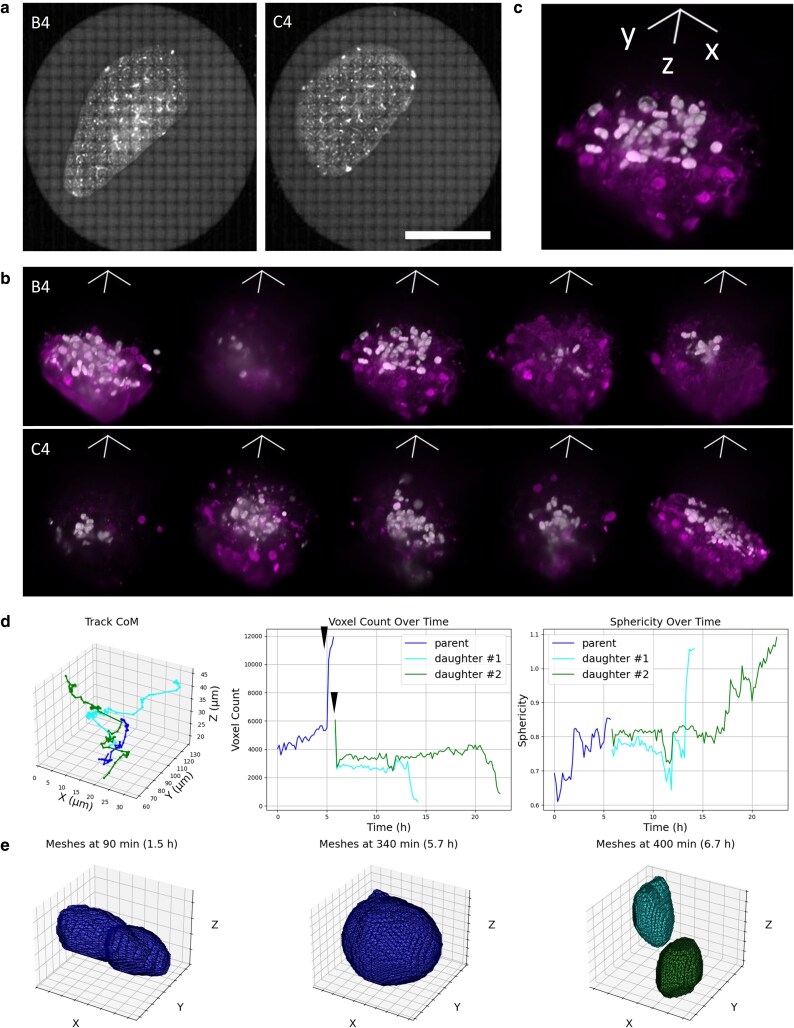
Time-lapse multi-FOV imaging of ex vivo tumor xenografts in precision-cut lung slices: H1975 cells expressing nuclear CFP were injected in the tail-vein of NSG mice and allowed to form tumors for 4 weeks before lung extraction, sectioning and imaging. 30 FOV were imaged at 10-min intervals over 144 timepoints. a) Exemplar tiled brightfield 60× imaging of two of the five lung slices. Scale bar 5 mm. b) 3D rendering showing the 5 FOV imaged in each of the two wells shown in (a). White, nucleus; magenta, E-cadherin; volume shown, 180 × 200 × 255 m^3^. c) Enlarged view of third FOV from well B4. d) Center of mass (left), nuclear ROI volume (center), and nuclear ROI sphericity (right) of one exemplar tracked cell from the FOV shown in (c) before division (blue—i.e. line between ∼0 to 6 hours) and its progeny after division (green and cyan, i.e. the two lines after ∼6 hours). e) Nuclear ROIs before (left and center, frames 9 and 34, *t* = 90 and 340 minutes) and after (right, frame 40, *t* = 400 min) division. All 3D rendering xyz scale bar lengths 50 µm.

To demonstrate the ability of dOPM to achieve quantitative readouts in time-lapse 3D imaging of arrays of samples, dOPM was used to image 300 μm thick precision-cut lung slices obtained from orthotopic tumors generated by tail-vein injection of human H1975 cells into mice. The H1975 cells expressed nuclear CFP, and E-cadherin antibody labeling added *ex vivo* provided structural context. Thirty FOVs were imaged at 10-min intervals and the results from 10 exemplar FOV are shown in Fig. 3b and Movie S4. Figure 3c shows a zoom-in of one FOV and the corresponding movie is shown on the left-hand side in Movie S5. The right hand side of Movie S5 highlights the track of a cell and its division over time, and the corresponding region of interest (ROI) center of mass, volume and nuclear sphericity are shown in Fig. 3d and e. The increase in ROI volume is consistent with nuclear envelope breakdown prior to mitosis and its reformation afterwards (black arrowheads, Fig. 3d).

### FUCCI readouts of cell-cycle stage

3D multi-FOV imaging of live murine NF1^−/−^; PTEN^−/−^; EGFRVIII (NPE) glioblastoma spheroids (26) expressing a FUCCI cell cycle reporter system (Cdt1-mCherry and Geminin-mVenus) (27) as well as a Histone H2B marker (mCerulean) and cytoplasmic GFP was performed using dOPM, with up to 10 spheroids imaged per well in 96-well plates.

**Fig. 4.**
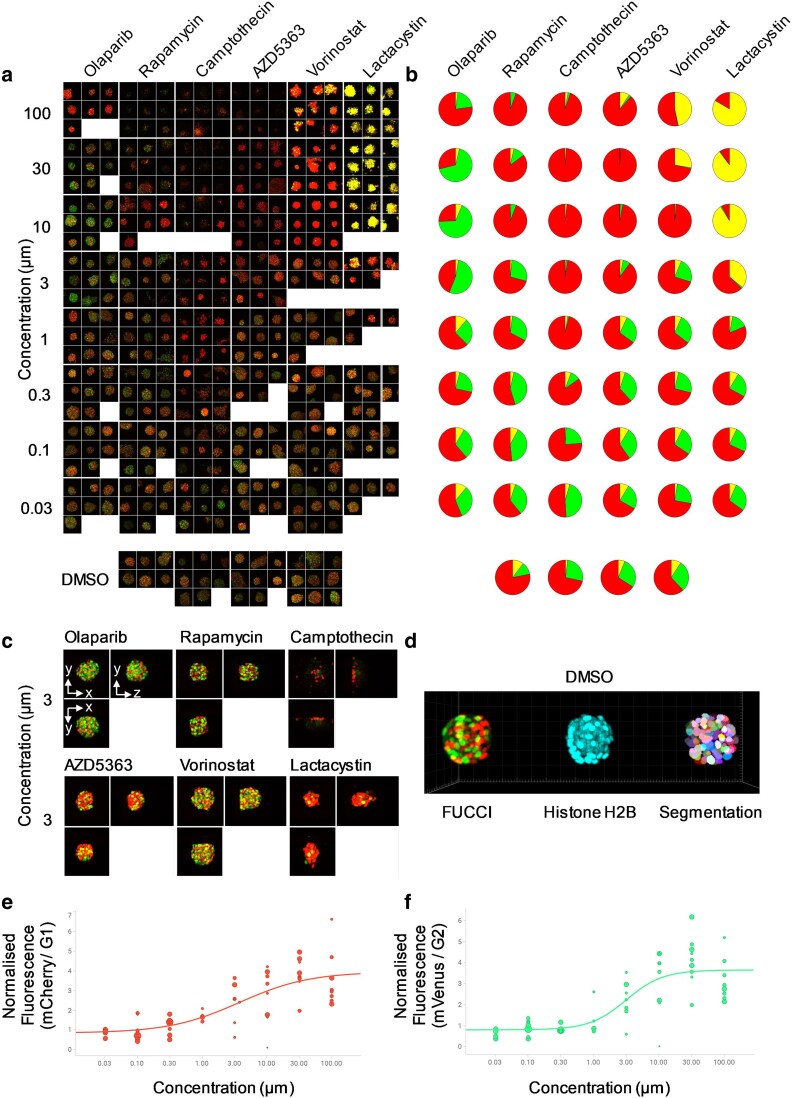
3D multi-FOV imaging of live murine NPE glioblastoma spheroids expressing the FUCCI cell cycle reporter and H2B marker (mCerulean) and treated with varying concentrations of six drug compounds. a) FUCCI MIPs acquired 24 h after treatment. Up to 10 spheroids were imaged in each well, FOV = 140 μm × 140 μm, with up to nine shown. b) Average proportions of individual nuclei classified into G2 (green), G1 (red), or S (yellow) for each well—for example, for highest concentration of Olaparib, G1>G2>S, and for the highest concentration of Lactacystin, S>G1>G2. c) Example orthogonal MIPs from spheroids treated with 3 μM of each compound tested. d) 3D rendering of a single spheroid showing the FUCCI (left), histone H2B (center) and segmentation of the nuclei used for analysis (right). Dose response of (e) the FUCCI Cdt-1 (mCherry) and (f) Geminin (mVenus) markers to Lactacystin. Fluorescence intensities in each channel were normalized to DMSO-treated controls and each data point represents an individual spheroid, with the size of the point proportional to the number of nuclei in the spheroid.

Spheroids were located using a semiautomatic prefind procedure, see Methods, and were exposed to a range of concentrations of 6 drugs, see Fig. 4a. The H2B channel was used to automatically segment the nuclei, see Fig. 4d center and right panels and Methods. Nuclear masks were then used to calculate the Cdt1-mCherry and Geminin-mVenus signal from each nucleus. This allowed classification of cell-cycle status for each individual cell, including those in S-phase, which is not possible from nonsectioning/whole-spheroid readouts. Average proportions of each cell-cycle stage for each well are shown in Fig. 4b. This enabled dose–response curves to be generated for each FUCCI reporter following drug treatment, see example shown in Fig. 4e and f for Lactacystin, a potent proteasome inhibitor that has previously been shown to modulate cell cycle progression (28, 29) but is also expected to stabilize both FUCCI reporters. We also calculated the industry-standard coefficient of variation per well for the average G1 and G2 intensities in the DMSO control wells, yielding values of 16 and 18%, respectively, which can be acceptable for a high-throughput screening assay (30). The EC50 values for the red and green signals are 3.6 μM and 3.0 μM, respectively.

### Stage-scanned dOPM

**Fig. 5.**
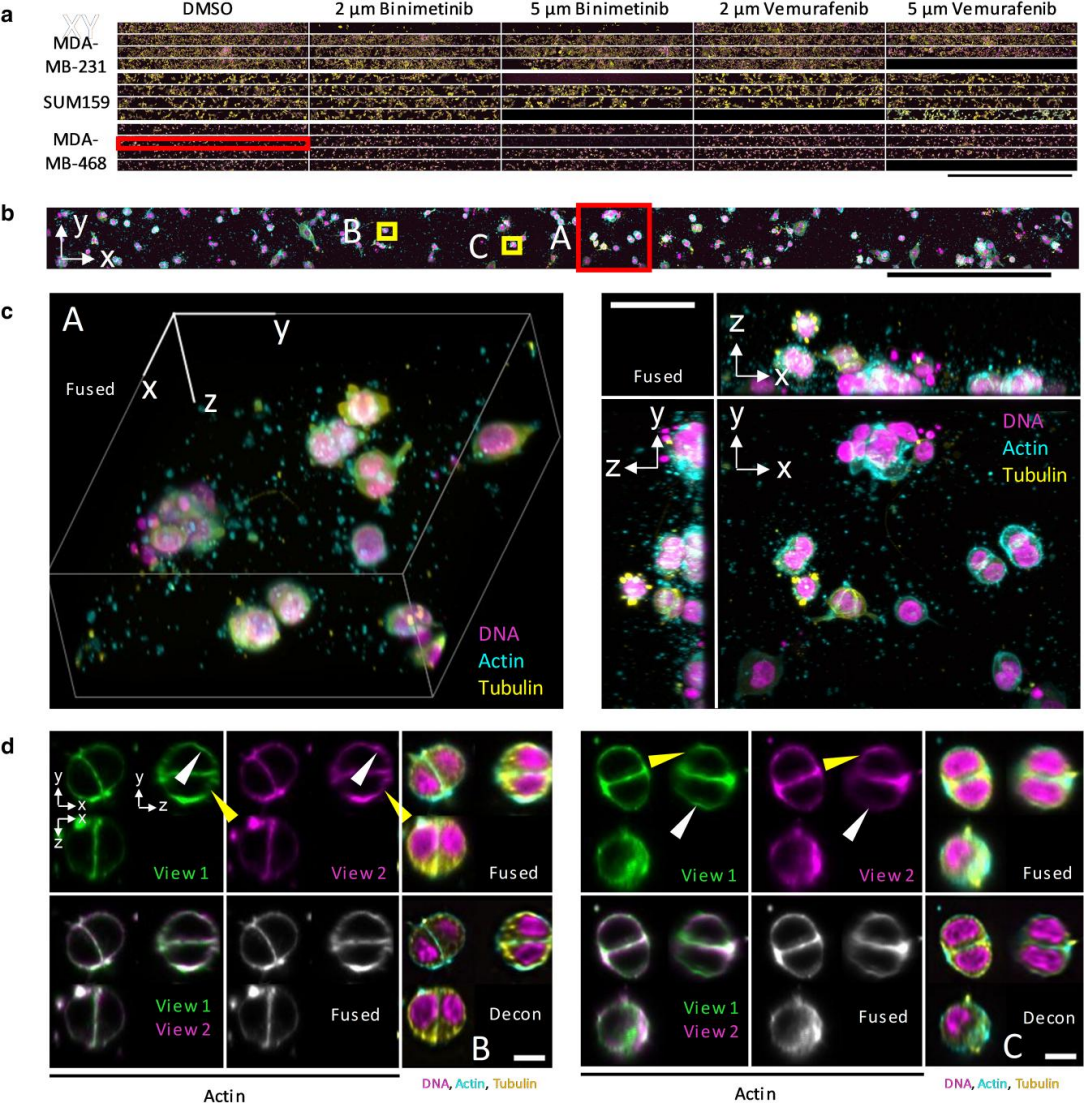
Fixed MDA-MB-231, SUM159, and MDA-MB-468 breast cancer cells grown in 3D in collagen in a 96-well plate. Cells treated with DMSO (control), 2 and 5 µM Binimetinib and 2&5 µM Vemurafenib. a) x–y MIPs of whole FOV for each well, top panel MDA-MB-231 cells, middle panel SUM159 cells and lower panel MDA-MB-468 cells. b) x–y MIP for well indicated highlighted by rectangular box overlay (2nd row of DMSO column for the MDA-MB-468 panel of wells) in (a). FOV shown is 178 × 3,147 ×m^2^. c) 3D rendering (left) and orthogonal MIPs (right) of region (A) shown in (b). FOV shown is 178 × 212 × 71 μm^3^. d) Orthoplanes of regions (B, left; C, right) shown in (b). Top left, view 1; top center, view 2; bottom left, overlay of view 1 and view 2; bottom center, fused volume. Top right fused and bottom right deconvolved orthoplanes showing all three channels. Scale bar in a) 2000μm, b) 500 μm, c) 50 μm, and d) 10 μm.

The dOPM system was used in stage-scanning mode to study human TNBC cells embedded in 3D matrices across extended FOVs in a 96-well plate. Three TNBC cell lines were treated with the BRAF inhibitor Vemurafenib and the MEK1/2 inhibitor Binimetinib at two different concentrations, alongside a DMSO vehicle control, see Fig. 5. The resulting data demonstrates the capability of the system to acquire 3D cell-shape data across multiple experimental conditions in a 96-well plate in ∼3.5 h, see Fig. 5a. Overall, 14,055 cells were imaged across 53 wells, see Table S2. Figure 5d shows two exemplar pairs of cells from this dataset for views 1 and 2 individually, the fused and deconvolved data. White and yellow arrowheads indicate regions of the membrane resolved clearly in views 1 and 2, respectively. The cell-shape information acquired here could for example be analyzed using the machine learning approaches applied previously to lower resolution (single-view) OPM data (31).

Stage-scanned imaging with stitching was also demonstrated by imaging patient-derived tumor organoids (PDO) in basement membrane extract, see Fig. S10 and Movie S6. This demonstrates the ability of the system to image an extended FOV (ultimately only limited by xy-stage scan range) of 826 × 1157 × 113 μm^3^ containing on the order of 20 organoids in 3 colors in 6 min—with the acquisition speed limited here by the Nikon NIS-Elements image acquisition software.

## Discussion and conclusions

### Spatial resolution

We measured the spatial resolution of fused dOPM imaging over a volume of 100 × 100 × 100 μm^3^. The lateral spatial resolution was found to be better for the 35° OPM angle, with median bead FWHM ± IQR of 0.29 ± 0.02 and 0.31 ± 0.03 μm in the x and y directions, respectively. There are two common ways of measuring the axial performance of a microscope: the axial FWHM of the point spread function (PSF) and the optical sectioning strength (32), which characterizes how the zero spatial frequency attenuates with defocus. For the 35° OPM angle, we measured the median axial bead FWHM to be 0.83 ± 0.21 μm, and the median sectioning strength to be 2.45 ± 0.18 μm (axial profile of laterally integrated bead signal) and 3.00 ± 0.14 μm (from a thin fluorescent sheet). For comparison, we have summarized the spatial resolution results from a range of relevant previously published systems, see Table S3.

With remote-refocusing systems, it is important to characterize how well the system performs away from the design focal plane of the primary objective (10, 33, 34). Considering systems with similar spatial resolution, see Table S3, the work by Sirinakis et al. (35) achieved a 274–315 nm lateral FWHM for a 50 μm lateral radius from the optical axis over 20 μm axially and the work by Ivanov et al. (36) achieved a 262–296 nm lateral resolution over a ∼100 × 120 μm^2^ FOV over 15 μm axially. The dOPM system reported here provides marginally lower spatial resolution, but we have characterized the dOPM resolution over a ∼5-fold larger axial range of 100 μm. The spatial resolution measurements reported here were performed for ideal samples of fluorescent spheres embedded in agarose. Real samples will exhibit absorption, scattering and refractive index variations that will inevitably affect the image quality. Example x–z and y–z images from complex biological samples illustrating the spatial resolution achieved at depths up to 100 μm are shown in e.g. Fig. 2d. We note that the system presented by Ivanov et al. (36) is capable of imaging over an axial range of 60 μm, but the system was characterized over a smaller range.

In single-view LSFM data, there is generally a “bad” direction to view the data (YZ in this article), i.e. the axial plane including the illumination beam propagation direction where shadowing artifacts are most visible and the poorer resolution in the direction perpendicular to the light sheet plane is most obvious. In this article, we have used fused dual-view OPM to demonstrate 3D imaging where these effects are less apparent and the spatial resolution in the three orthoplanes is more similar (Fig. 1d).

In the future, it will be possible to make use of adaptive optics OPM to correct for sample induced aberrations, such as has been demonstrated advantageously on an upright OPM system (37), in dOPM and on inverted microscopes for use when imaging arrays of samples in multiwell plates.

### Collection efficiency

The folded OPM approach employed in dOPM has reduced transmission for fluorescence collection due to the PBS in the collection path. For an ideal dOPM system the transmission is 50% for completely depolarized fluorescence from rapidly tumbling fluorophores assuming low-NA detection, and the same result is obtained in our high-NA simulation (Fig. S7c, column 2, 0°). In the case of a static fluorophores, the theoretical low-NA transmission is 75%, and in our vectorial simulation this is calculated to be 71% for the 1.2 NA water immersion lens used in the dOPM setup (Fig. S7f, column 2, 0°).

We measured the transmission of our system for an OPM angle of 35° relative to the signal after TL1 and obtained 19 and 23% for the cases of rapidly tumbling and static fluorophores respectively, which includes optical losses due to the double pass of O2/3 and the PBS. These measurements were carried out using emission from fluorescent beads that provide an accurate model for the distribution of intensity and polarization states in the back focal plane of O1.

Our transmission measurements can be considered in the context of OPM systems employing a high-NA low-working-distance tertiary objective. The work by Yang et al. (13) measures the transmission of O2 and O3 in their system and reports values in the range 62–73%, but the diameter of the laser beam used was only given approximately and variations will affect how much the pupils of O2–3 clip the beam. The work by Sapoznik et al. (15) reports transmission measurements for laser scanning of 41% and stage-scanning of 53% using a HeNe laser beam chosen to match the diameter of the pupil of O1. Our method using fluorescence emission from beads automatically produces the correct beam distribution in the pupil of O1.

### Photobleaching measurements

In our measurement of the photobleaching of dOPM relative to wide-field fluorescence z-stack imaging, we chose to normalize the comparison by setting the total detected signal level for a point object in focus to be the same for both methods. This was chosen as the dominant Poisson (shot) noise on many quantitative measurements, e.g. total signal measured over a cell or over a particular sub-cellular organelle, will be determined by the total signal level recorded over the given region. In addition, the same primary objective lens and identical cameras were used in the comparison. Ultimately, photobleaching—which is often considered a proxy for photodamage—can be assessed for the specific quantitative metric that is of interest in a given experiment. But as many quantitative measurements depend on the Poisson (shot) noise on the detected signal, normalizing the bleaching comparison to total detected signal for a point object can be useful depending on the situation.

### Biology applications

We have demonstrated the ability of dOPM to provide high spatial resolution images in 3D over multiple FOVs in a range of biologically relevant samples including arrays of patient-derived organoids and arrays of ex vivo lung slices. This includes the ability of the system to image relatively rare 3D events, such as the opening and subsequent closure of a lumen within an organoid and the migration of organoids within gels (Fig. 2). We have demonstrated the ability of dOPM to track cell division and quantify nuclear shape in single cells in arrays of ex vivo tissue slices (Fig. 3). We have shown that dOPM enables classification of cell-cycle status at the single-cell level within live glioblastoma spheroids expressing the FUCCI cell-cycle state reporter and can produce sigmoidal dose–response curves against each cell-cycle endpoint following drug treatment in standard multiwell imaging plates (Fig. 4). Furthermore, the use of stage-scanned image acquisition allowed 3D imaging of 14,055 cells in collagen gel over multiple experimental conditions (three cell lines, two drugs, three drug concentrations, Fig. 5).

### Summary

3D imaging at scale with sub-cellular resolution and quantitative readouts in multiwell plates is becoming increasingly desirable as biomedical scientists seek to perform studies in more biologically realistic specimens compared to 2D cell culture. We have demonstrated that dOPM causes significantly less photobleaching compared to wide-field image stacks when using our standard image acquisition parameters and therefore less photobleaching compared to an ideal spinning-disk confocal system using the same objective lens. Compared to OPM systems employing high-NA low working distance tertiary objectives, the fluorescence collection efficiency of dOPM is lower. However, the spatial resolution achieved with dOPM is similar and we have demonstrated that this resolution is approximately maintained over an axial range of 100 μm, which is a ∼5-fold greater range than reported in previous OPM studies with similar spatial resolution, and in all cases comparing data obtained from sub-resolution fluorescent spheres in a homogenous medium. dOPM has a relatively compact optical system, there is only one moving part in the system (PIMAG translation stage) for both switching between views and remote scanning, there is no need to purchase a tertiary objective, which can be costly, the system is spectrally flexible as it does not require a dichroic beam-splitter, it enables tuning the light-sheet illumination and detection angle by only a swap of a mirror prism unit, and implements dual-view imaging for reducing shadowing artifacts and to achieve a more isotropic spatial resolution. We have demonstrated dOPM for a wide range of biological applications and believe that it achieves a practically useful balance of the performance-cost-complexity trade-off in microscope design.

## Methods

### Optical system

The dOPM optical system is shown in Fig. S5. It is based on that described in reference (21) but configured with a 60×/1.2NA Nikon water immersion primary objective. Full details of all optical components are given in Table S4. Light from an Omicron LightHUB equipped with five laser lines is expanded 8.3× by the telescope formed by L1 and L2, and the beam diameter is set by a 6 mm diameter iris diaphragm (ID) placed after L2. Achromatic half and quarter-wave plates HWP3 and QWP3 are then used to control the polarization state. Additionally, HWP1&2 and QWP1&2 are placed in the 642 and 445 nm light paths. These allow polarization aberrations generated when reflecting off M3 or M7 to be corrected separately for these two excitation wavelengths. The other three wavelengths (488, 515, and 561 nm) are sufficiently close together in wavelength that the polarization aberrations can be corrected together using HWP3 and QWP3. In the future, it may be possible to avoid these polarization aberrations using mirror coatings designed to minimize such effects (38).

The beam is then split into two by a nonpolarizing beam splitter, with each path generating the illumination light sheet for one of the two views. Each path includes protected-silver mirrors (M1,2,4,5,6), a cylindrical lens (CL1 or CL2) and microscope objective (O5 or O6) to form a light sheet in the x–y plane perpendicular to the optical axis of O2/3, which is a 50×/0.95 Olympus air immersion objective. To achieve an OPM angle of 35°, a prism mirror (M3 or M7) with its normal at 17.5° to the optical axis of O2/3 is used. For an OPM angle of 45° the prism mirror normal is at 22.5° to the optical axis of O2/3. Mirrors M3 and M7 are mounted side by side on a common mount on a voice-coil stage (PI, V-522.1AA) oriented to translate the two mirrors in the y-direction, which is perpendicular to the optical axis of O2/3. The voice-coil stage allows both scanning of the light sheet through the sample for each view and switching between view 1 (Fig. S5a) and view 2 (Fig. S5b).

Objective O2/3, a pair of tube lenses (TL2 and TL1) and objective O1 are configured to give an overall magnification of 1.333 from sample space in the front focal plane of O1 to the remote space in the front focal plane of O2/3 to meet the condition for remote refocusing described by Botcherby et al. (9, 33, 34). To achieve this, TL2 is formed by a pair of achromatic doublets whose separation is adjusted to achieve the desired overall magnification. To increase the long-term stability of the optical alignment, O2/3 is fitted with a heater set to maintain a constant temperature (set a few degrees above the typical ambient temperature).

After passing through O2/3, an achromatic quarter-wave plate (QWP4) converts the excitation light to be vertically linearly polarized and it then transmits through a PBS. The excitation light is then relayed to the sample (S) via TL2, TL1, and O1.

Fluorescence generated in the sample by the light-sheet illumination is collected by O1, relayed by TL1 and TL2 and transmission through the PBS to O2, reflected by the prism mirror for the current view (M3 or M7), reflected by the PBS following double-passing QWP4 and an image is formed by TL3 onto the camera (sCMOS1). Emission filter EM1 is mounted on a motorized filter wheel (LB10-3, Sutter) and used to define the detection spectral bandpass.

Details of the image acquisition process, image deskewing and registration, and fluorescence bead segmentation and quantification are given in the Supplementary Methods. To show spatial co-registration of 3 spectral channels, see Fig. S11.

### Geometric collection efficiency calculation

First, the collection solid angle of the dOPM system, $\Omega_{\mathrm{OPM}}$, was calculated numerically by the area of intersection of the collection cones of O1, O2, and O3 on the surface of a unit sphere, see Fig. S12 where the collection cones of O1, O2, and O3 are shown in red, green and blue respectively, with the overlap of the collection cones of O2 and O3 shown in yellow. The calculated latitudinal and longitudinal NAs for the system are shown in Table S1.

The ideal transmission efficiency for fluorescence of a double pass of the PBS and QWP4, $T_{\mathrm{PBS}\;\mathrm{fluo}}$, was then taken to be 50% in the case of fully depolarized fluorescence emission from rapidly tumbling fluorophores and 75% for the case of partially polarized emission from static fluorophores. This is derived from Lakowicz (39), Equation 10.1 for steady-state anisotropies of 0 and 0.4, and is only valid for low NA.

The optical transmission efficiency for linearly polarized light double-passing O2/3 and the PBS was then estimated. The transmission for a single-pass of O2/O3 stated by the manufacturer is *T*_O2/3_ = 0.9, and the experimentally measured transmission of collimated linearly polarized on-axis light for a wavelength of 532 nm double-passing through the PBS (i.e. through PBS, QWP4, Thorlabs PF05-03-P01 protected silver mirror at normal incidence, QWP4, PBS) was *T*_PBS_ = 0.8. Overall, this gives a transmission for the optics of $T_{\mathrm{optics}}=T_{O2/3}^{2}T_{\mathrm{PBS}}$.

The RCE is defined as the collection efficiency of the dOPM system relative to the collection solid angle of O1, $\Omega_{O1}$,


$$\mathrm{RCE}=\frac{\Omega_{\mathrm{OPM}}}{\Omega_{O1}}T_{\mathrm{optics}}T_{\mathrm{PBS}}.$$

### Vectorial raytracing and collection efficiency calculation

A vectorial raytracing simulation written in Python (see Ref. (40)) was used to calculate the light collection efficiency and light intensity distributions in the exit pupil of O3 for a dOPM system. A cone of 15,000 rays was initially generated over 2π steradians to overfill the pupil of O1, see Supplementary Simulation Methods (SSMs) Figs. S1 and S2. In an approach similar to that used by Kim et al. (41) and Sommernes et al. (42), Jones matrices were calculated for each ray to include the effect of all optical elements in the dOPM system, listed in SSMs Table S1, and to vectorially trace rays and electric fields from the source to the pupil of O3. The source was realized by an ensemble of 7,500 electric dipole-emitter point sources with their orientations filling a hemisphere and generated using the Fibonacci spiral method, see SSMs. For each dipole source, the initial electric field for each ray within the cone of rays was calculated and the electric field at the pupil of O3 was then calculated using the Jones matrix precalculated for that ray, see SSMs Fig. S3.

In these simulations, each ray had an associated wavevector and electric field vector, see SSMs Fig. S4. The Jones matrices for lenses apply rotations in the meridional plane, requiring a meridional transform matrix, while those for linear polarizers, waveplates, and the tilted mirror operated in Cartesian lab coordinates, see SSMs Fig. S5. Unlike previous vectorial raytracing calculations (43), the effect of the tilted mirror surface on the electric field and the effect of the mirror coating layer was included. The amplitude and phase changes of the electric field components parallel and perpendicular to the reflection plane (p and s, respectively) were calculated using the complex reflectivity derived from thin film reflection theory (44), see SSMs Fig. S6. Householder matrices were then used to calculate the direction of the reflected electric field and rays. More detailed descriptions of the Jones matrix for each element are given in the SSMs.

The simulation results were then scaled by *T*_optics_ in the same way as the geometric collection efficiency calculation.

### dOPM collection efficiency measurements

To assess the collection efficiency of the dOPM system relative to that of a widefield epi-fluorescence microscope with identical primary objective (O1) and tube lens (TL1), we measured the total fluorescence signal from isolated 200 nm diameter fluorescence beads under epi-illumination. The experimental setup used is shown in Fig. S6. A single spatially isolated bead was positioned in the front focal plane and in the center of the FOV of O1. The epi-fluorescence signal was then recorded using LED illumination via a dichroic filter cube (EX, D, EM2) and TL1, with emission being reflected from a switching mirror (SM) onto the epi-detection camera (sCMOS2) (epi-before image). Without changing any other settings, the switching mirror (SM) was removed, and a second image was acquired on the dOPM detection camera (sCMOS1) (dOPM image).

This procedure was performed first for unpolarized excitation to provide depolarized fluorescence emission, which models the case of rapidly tumbling fluorophores. The procedure was then performed again with a polarizer (P) inserted into the epi-illumination path; this models the case of dOPM excitation with linearly polarized illumination with the electric field vector in the plane of the illumination light sheet exciting static fluorophores, which results in partially polarized fluorescence emission. The whole process was repeated for 3 fluorescent beads.

The total fluorescence signal from each bead (*S*) was found by summing the fluorescence signal over a square ROI chosen to include at least 99% of the bead energy (calculated assuming an Airy distribution). Background signal (*B*) was then measured from a square ROI located adjacent to the bead ROI. The relative dOPM collection efficiency *η* was then calculated as the ratio of the background corrected signals using


(1) $$\eta =\frac{S_{\mathrm{dOPM}}-B_{\mathrm{dOPM}}}{S_{\mathrm{EPI}}-B_{\mathrm{EPI}}}.$$

### Preparation of biological samples and imaging protocols

See Supplementary Methods including Table S5.

Ex vivo tumor xenografts in precision-cut lung slices: The Francis Crick Institute Animal Welfare and Ethical Review Body and UK Home Office authority provided by Project License 0736231 approved all animal model procedures.

### dOPM photobleaching compared to widefield imaging for equal fluorescence signal

See Supplementary Methods including Table S6.

## Supplementary Material

pgaf370_Supplementary_Data

## Data Availability

Datasets reported in this manuscript are available from Bioimage Archive at https://www.ebi.ac.uk/biostudies/bioimages/studies/S-BIAD2314. The software used for collecting and processing these datasets is available via our GitHub wiki https://github.com/ImperialCollegeLondon/oblique-plane-microscopy/wiki.
