# Supplementary material for: High content 3D imaging by dual-view oblique plane microscopy
Source: PNAS Nexus. 2025 Nov 26;4(12):pgaf370. doi: 10.1093/pnasnexus/pgaf370 (PMC12680133; doi:10.1093/pnasnexus/pgaf370)
Supplement: pgaf370_Supplementary_Data [file pgaf370_supplementary_data.zip › PNASNEXUS-PNASNEXUS-2025-00994R-s01.pdf]

High Content 3D Imaging by Dual-View Oblique Plane Microscopy

Sparks et al.

Supplementary Material

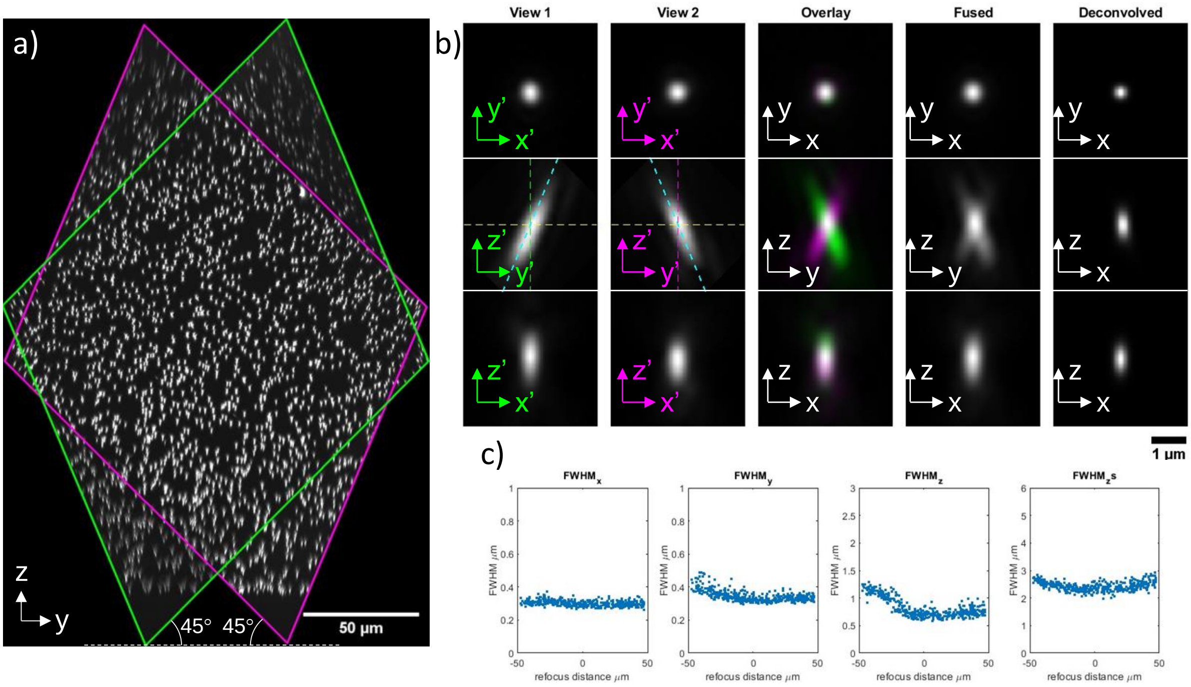

Supplementary Figure 1: dOPM imaging of 100 nm fluorescent beads embedded in 3D in agarose obtained for a 45° OPM angle. (a) MIP of fused bead volume along x direction. (b) Orthoplanes through a representative bead for view 1, view 2, the fusion of views 1 and 2 and deconvolution of views 1 and 2. (c) Plots of fused bead image FWHM in the x, y and z directions for all beads within a 100^3 μm^3 volume as a function of z position within volume. The righthand plot shows the results for the z sectioning.

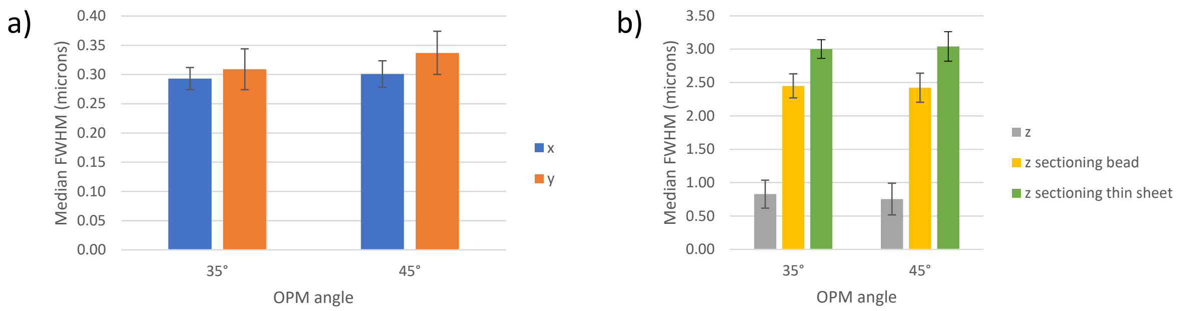

Supplementary Figure 2: (a) Plots of the median bead FWHM for the fused data in the x and y directions over the full 100^3 μm^3 volume for both 35° and 45° OPM angles. (b) Same as for (a) but showing the bead image FWHM in z, the z sectioning measured from the 100 nm bead volume and the z sectioning measured from a thin fluorescent sheet. Error bars in (e)&(f) show the IQR. The 95% confidence interval on the median is given in Table 1.

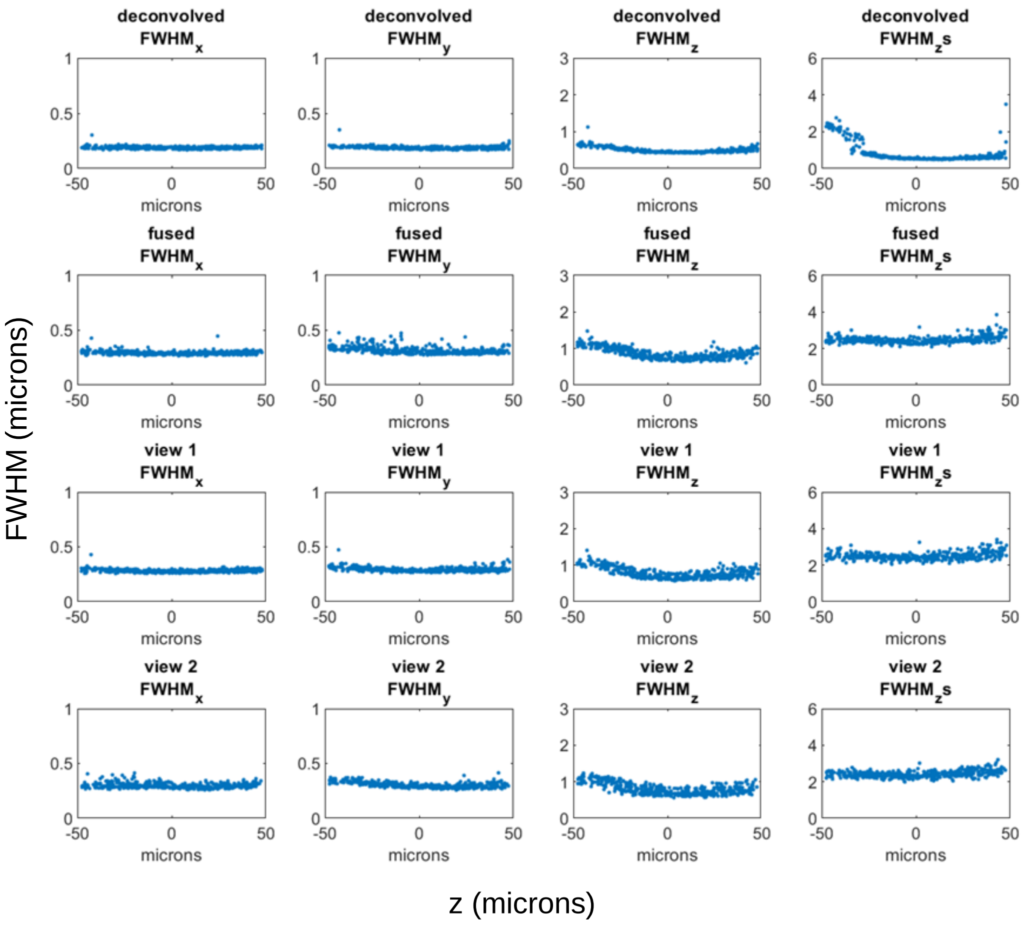

Supplementary Figure 3: Plots of bead image FWHM in the x,y and z directions for all beads within a 100^3 μm^3 volume as a function of z position within volume for an OPM angle of 35°. Each point represents the value from a single bead. The columns from left to right show the FWHM in x, y and z and the z sectioning respectively. The rows from top to bottom show results for deconvolution, fusion, view 1 only and view 2 only respectively.

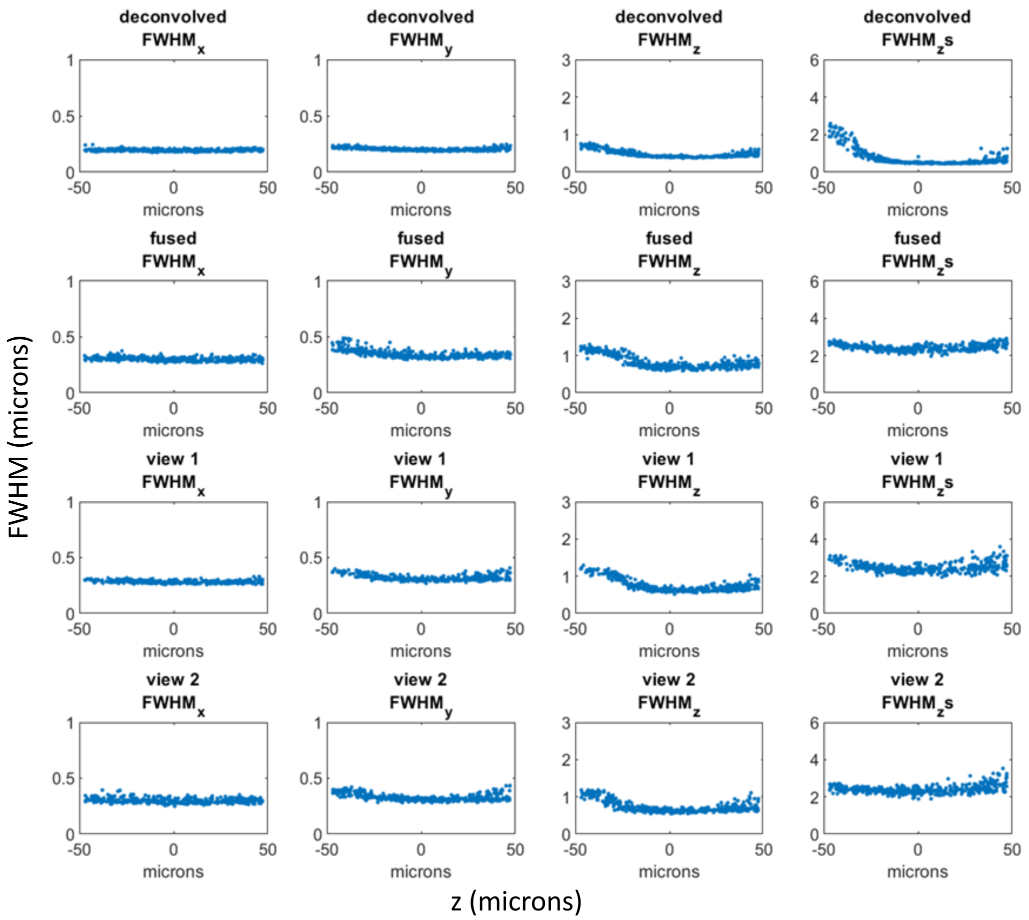

Supplementary Figure 4: Plots of bead image FWHM in the x,y and z directions for all beads within a 100^3 μm^3 volume as a function of z position within volume for an OPM angle of 45°. Each point represents the value from a single bead. The columns from left to right show the FWHM in x, y and z and the z sectioning respectively. The rows from top to bottom show results for deconvolution, fusion, view 1 only and view 2 only respectively.

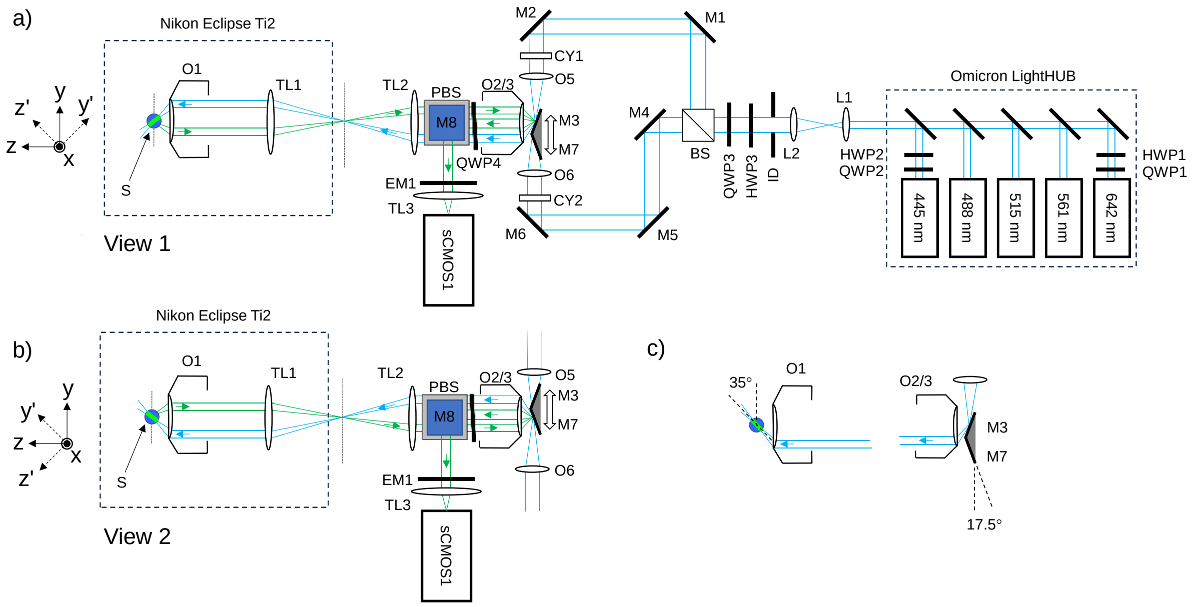

Supplementary Figure 5: dOPM imaging system. (a) Mirror assembly M3&M7 positioned so that M3 is at the focus of O2/3 to achieve a first view angle (view 1) of the sample. (b) M7 at the focus of O2/3 to give second view angle (view 2) of the sample. HWP, half-wave plate; QWP, quarter-wave plate; L, lens; ID, iris diaphragm; BS, non-polarising beam splitter; M, mirror; CY, cylindrical lens; O, objective lens; PBS, polarising beam splitter; TL, tube lens; S, sample; EM, emission filter. The region within the dashed black boxes is contained within a Nikon Eclipse Ti2 microscope frame. Laboratory coordinates are labelled x,y,z. The coordinate system for the dOPM views are labelled x', y', z'. For both views, directions x and x' are parallel. (c) Illustration showing the relationship between the M3/7 mirror angle and the light sheet illumination and view angle.

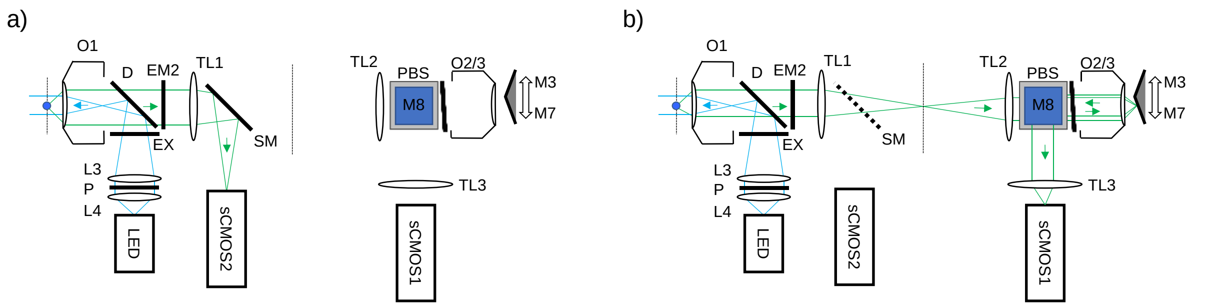

Supplementary Figure 6: Experimental configuration used for measuring the relative collection efficiency of the dOPM optics compared to epi-fluorescence imaging. (a) Shows the configuration used for the first step, where the total energy from single bead is measured using epi-fluorescence imaging both with and without polariser P in the illumination path. (b) Shows the second step, where the total energy from the same single bead as part (a) is measured with dOPM imaging under same illumination and again with and without polariser P. SM, switching mirror.

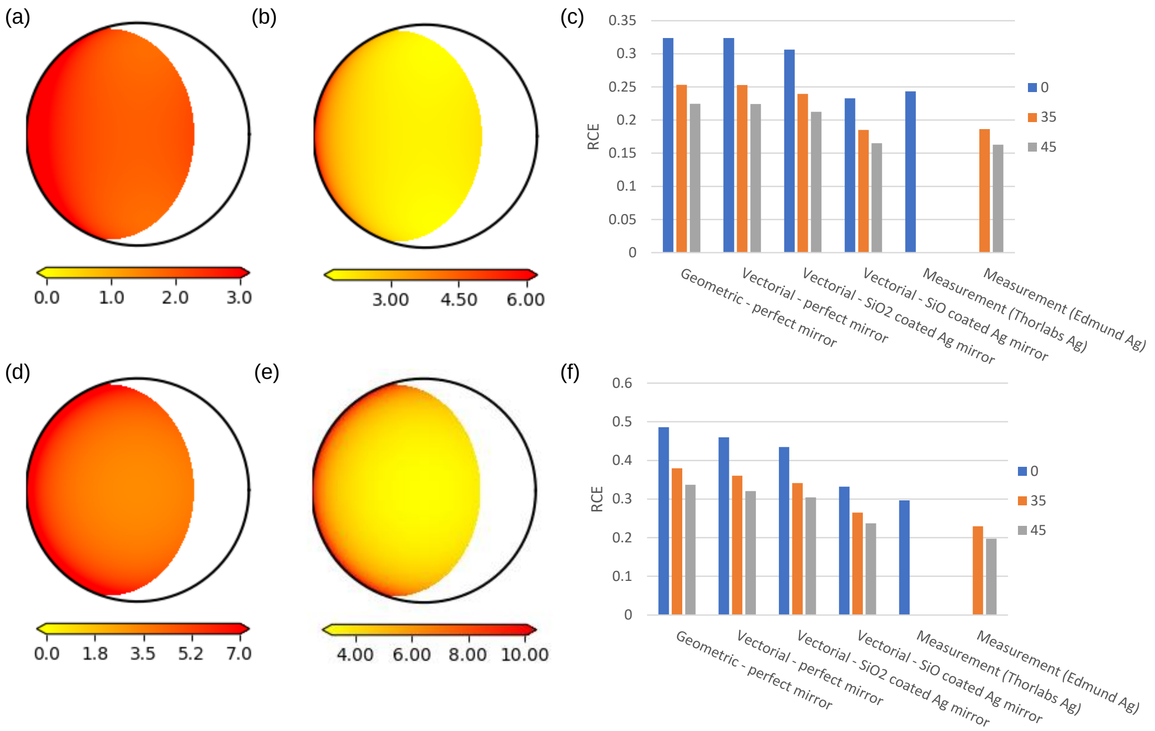

Supplementary Figure 7: Results from simulations and experimental measurements of dOPM fluorescence collection efficiency. (a-c) Data for the case of unpolarised fluorescence emission corresponding to fluorophores tumbling on a timescale much faster than their fluorescence lifetime. (d-f) Data for case of static fluorophores. (a&d) simulations for an OPM angle of 35° of the intensity distribution in back focal plane of O3 obtained from vectorial ray tracing, with the false-colour scale starting from zero. (b&e) same as (a&d) but with the false-colour scale adjusted to match the range of the data. (c&f) Plots of simulated and experimentally measured collection efficiency data for OPM angles of 0, 35 and 45° for a range of conditions, see main text for further description. RCE, relative collection efficiency compared to widefield epi-fluorescence imaging with same primary microscope.

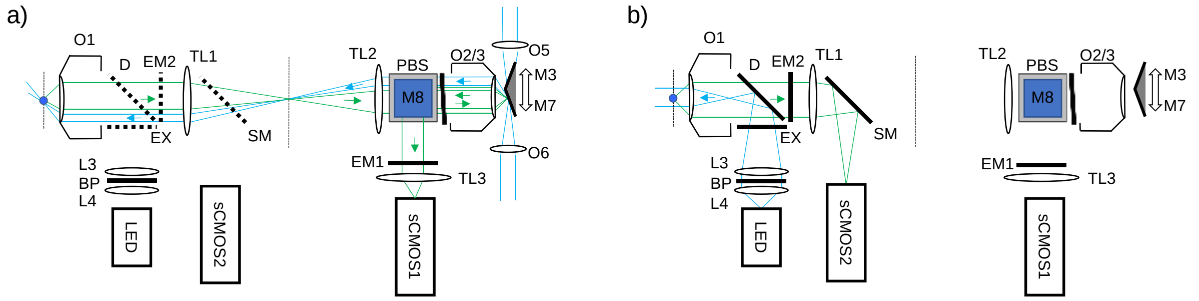

Supplementary Figure 8: Experimental configuration used to set dOPM and widefield epi-illumination excitation intensities for the dOPM photobleaching comparison. (a) Experimental configuration used to excite a single isolated 200 nm fluorescent bead with dOPM. (b) Experimental configuration used to excite same single isolated bead with widefield epi-fluorescence illumination. Only change to system compared to (a) is insertion of the epi-fluorescence cube (EX, D, EM2) and insertion of the switching mirror (SM). EM1 and EM2 are identical.

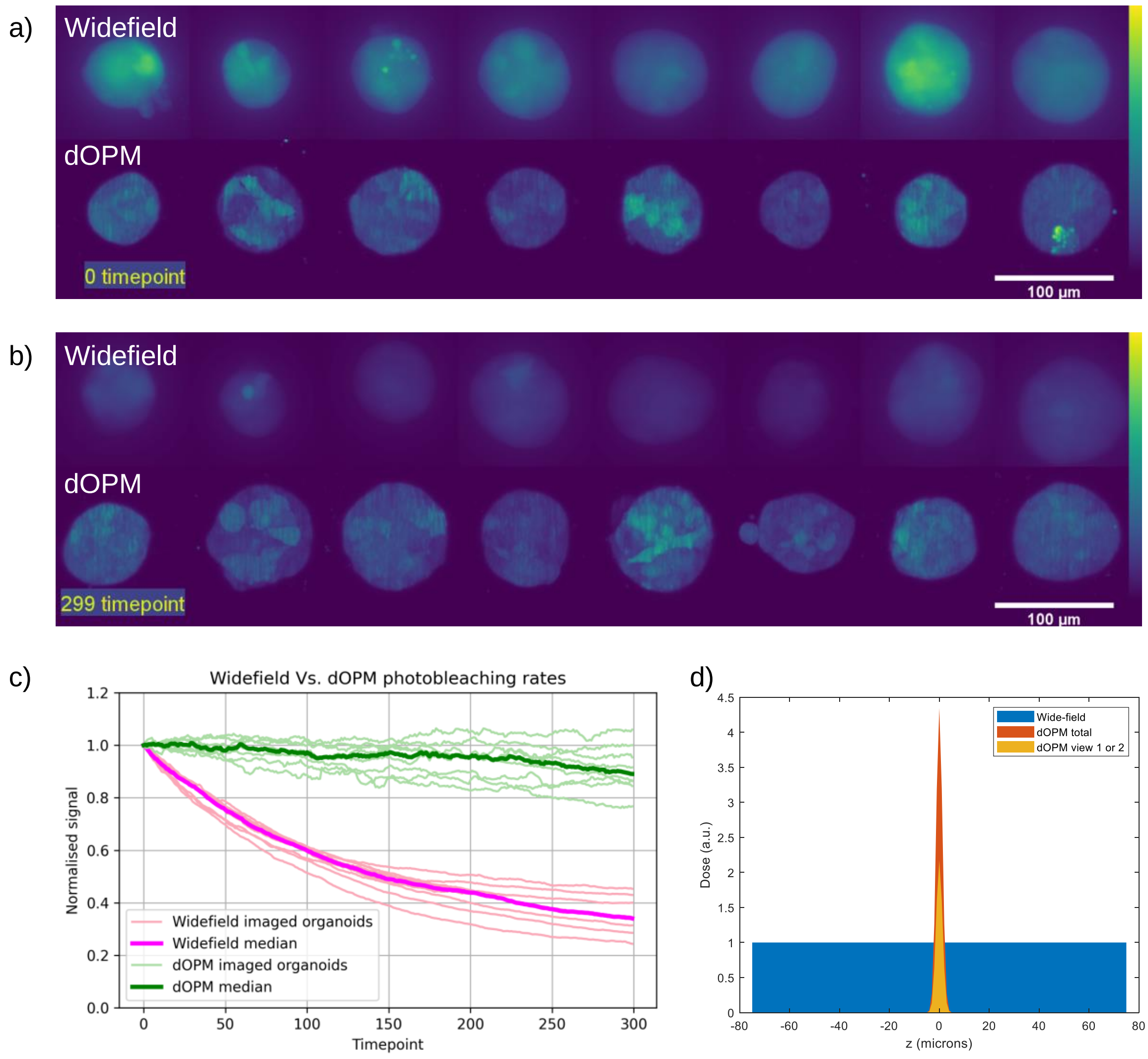

Supplementary Figure 9: dOPM photobleaching compared to epi-fluorescence imaging for equal fluorescence signal for a point object over 300 acquired image volumes. (a) x-y MIPS at timepoint 0 for 8 widefield epifluorescence imaging field of view (FOV) (top) and 8 dOPM FOV (bottom). (b) Same as (a) but for timepoint 299. (c) Normalised fluorescence signal for each spheroid over time (feint lines) and median signal for widefield and dOPM (bold lines). (d) Calculated light dose to sample for a stack of 151 images for: widefield epi-fluorescence imaging (blue), single-view dOPM imaging with one FOV (red), and for dual-view dOPM imaging (orange). The area under each curve represents the total light dose of an image stack.

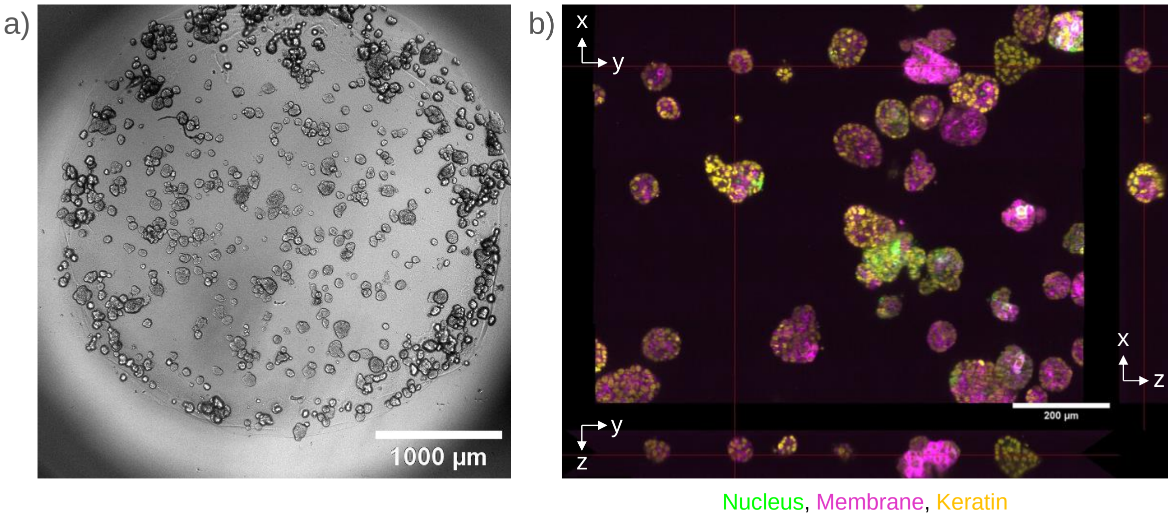

Supplementary Figure 10. Stage-scanned dOPM imaging of a fixed GS605 patient-derived tumour organoids (PDO), CRISPR knock-in engineered with Keratin20-iRFP fusion protein and lentiviral transfected with Histone-H2B-EGFP and CAAX-mCherry fusion proteins, in a 96-well format. (a) Brightfield image acquired at 4× magnification showing organoids embedded in a dome of basement membrane extract (BME) within a single well. (b) Resliced two-view fused stage scanned dOPM from 5 overlapping, 1 mm volumetric stripes scanned in y-direction and stitched together for final dimensions of 826×1157×113 μm^3.

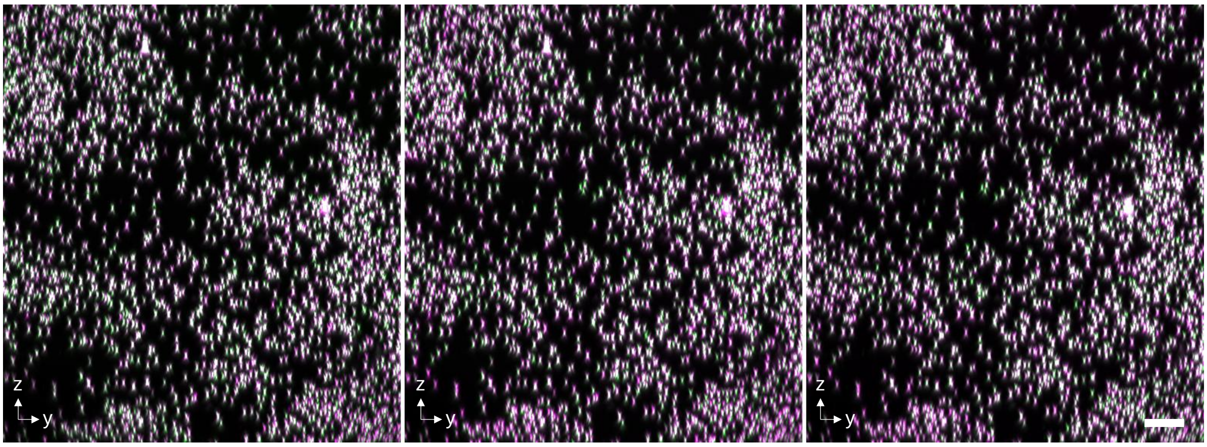

Supplementary Figure 11: Spatial co-registration of 3 spectral channels achieved using default co-registration settings of the Multi-View Fusion Plugin. Images are MIPs along x axis. Labels underneath each panel indicate the false-colour used for each channel as ex/em wavelengths in nanometres. The three panels show all permutations of pairs of the three spectral channels. Scale bar 10 µm.

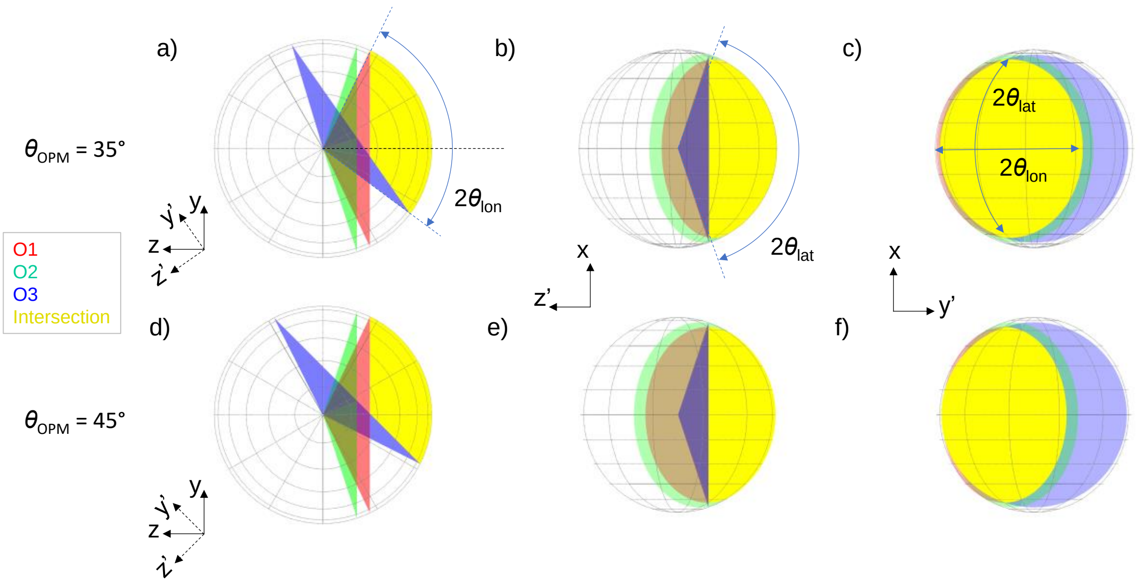

Supplementary Figure 12: Collection cones for dOPM at OPM angles of 35° and 45°. (a-c) θ_{OPM} = 35°. (d-f) θ_{OPM} = 45°. (a&d) z-y view, (b&e) z'-x view, i.e. perpendicular to detection direction and (c&f) y'-x view. The collection cones of O1, O2, O3 and the intersection are shown in red, green, blue and yellow respectively. The total latitudinal and longitudinal collection angles, 2θ_{lat} and 2θ_{lat} respectively, are indicated in (a-c). The corresponding NAs for these systems are tabulated in Supplementary Table 1.

Supplementary Table 1: Summary of calculated longitudinal and latitudinal NAs of the dOPM system as a function of OPM angle. Table also includes parameters relevant to the characterisation of the system spatial resolution. This information is also reported for a subset of systems reported below in Supplementary Table 3 to aid comparison.

	This work				Sirinakis et al. (1)	Ivanov et al. (2)
O1	60x/1.2 water				100x/1.35 silicone	100x/1.35 silicone
O2	50x/0.95 air				40x/0.95 air	40x/0.95 air
O3					AMS-AGY v1	AMS-AGY v1
θ_{OPM} (°)	30*	35	40*	45	28	30
Angle between dOPM views (°)	60	70	80	90		
λ_{em} (nm)	525				525	525
NA_{lat}**	1.2	1.2	1.2	1.2	1.33	1.33
NA_{lon}**	1.06	1.03	0.99	0.95	1.28	1.27
Effective pixel size for spatial resolution measurement (nm)	86				116	62
Data preprocessing	Single-view deskewed and dual-view deskewed fused				Single-view deskewed	Raw and single-view deskewed

* Not used for experiments, provided for context

** Calculated from stated specification of O1-3 and θ_{OPM}

Supplementary Table 2: Summary of cell types, drug treatment and number of cells detected on (<20 µm) and off (≥ 20 µm) the coverslip (in axial direction), together with the acquisition time for each well for the data shown in Figure 5. Description of the image segmentation method used is given below in the Supplementary Methods section Stage-scanned imaging of fixed MDA-MB-231 cells.

			Axial position relative to coverslip			
Well	Cell Type	Condition	On (<20 µm)	Off (≥ 20 µm)	Total	Acquisition time (minutes)
B4	231 cells	DMSO	480	34	514	4
B5	231 cells	Vemu 2uM	306	38	344	4
B6	231 cells	Vemu 5uM	382	58	440	4
B7	159 cells	Vemu 2uM	180	23	203	4
B8	159 cells	Vemu 5uM	132	19	151	4
B9	159 cells	DMSO	121	23	144	4
B10	231 cells	Bini 2uM	132	29	161	4
C2	231 cells	Bini 2uM	394	22	416	4
C3	231 cells	Bini 5uM	374	35	409	4
C4	231 cells	DMSO	377	36	413	4
C5	231 cells	Vemu 2uM	365	32	397	4
C6	231 cells	Vemu 5uM	341	53	394	4
C7	159 cells	Vemu 2uM	159	14	173	4
C8	159 cells	Vemu 5uM	113	8	121	4
C9	159 cells	DMSO	179	27	206	4
C10	231 cells	Bini 2uM	126	30	156	4
C11	231 cells	Bini 5uM	114	39	153	4
D2	231 cells	Bini 2uM	311	34	345	4
D3	231 cells	Bini 5uM	451	32	483	4
D4	231 cells	DMSO	418	46	464	4
D5	231 cells	Vemu 2uM	360	44	404	4
D6	231 cells	Vemu 5uM	304	55	359	4
D7	159 cells	Vemu 2uM	94	13	107	4
D8	159 cells	Vemu 5uM	116	18	134	4
D9	159 cells	DMSO	109	27	136	4
D10	231 cells	Bini 2uM	140	18	158	4
D11	231 cells	Bini 5uM	157	13	170	4
E2	231 cells	Bini 2uM	345	20	365	4
E3	231 cells	Bini 5uM	304	58	362	4
E4	231 cells	DMSO	379	23	402	4
E5	231 cells	Vemu 2uM	372	41	413	4
E8	159 cells	Vemu 5uM	146	12	158	4
E9	159 cells	DMSO	97	25	122	4
E10	231 cells	Bini 2uM	135	6	141	4
E11	231 cells	Bini 5uM	110	18	128	4

F2	468 cells	DMSO	123	39	162	4
F3	468 cells	DMSO	125	46	171	4
F4	468 cells	Bini 2uM	145	66	211	4
F5	468 cells	Bini 2uM	170	83	253	4
F6	468 cells	Bini 5uM	187	50	237	4
F8	468 cells	Vemu 2uM	193	86	279	4
F9	468 cells	Vemu 2uM	229	62	291	4
F10	468 cells	Vemu 5uM	193	64	257	4
F11	468 cells	Vemu 5uM	241	61	302	4
G2	468 cells	DMSO	186	68	254	4
G3	468 cells	DMSO	158	54	212	4
G4	468 cells	Bini 2uM	153	68	221	4
G5	468 cells	Bini 2uM	166	59	225	4
G6	468 cells	Bini 5uM	173	71	244	4
G7	468 cells	Bini 5uM	199	62	261	4
G8	468 cells	Vemu 2uM	191	57	248	4
G9	468 cells	Vemu 2uM	244	79	323	4
G10	468 cells	Vemu 5uM	200	58	258	4
				Total	14,055	212

Supplementary Table 3: Comparison of spatial resolution measurements in previously published OPM systems. Differences in the measurement methodology are summarised in the righthand three columns, which must be taken into account when making comparisons between the different results.

	PSF (nm)			Region over which PSF measured (µm)			n_{beads}	d_{bead} (nm)	PSF measurement (raw/deskewed)	PSF measurement method	Notes
Paper	X	Y	Z	X	Y	Z					
This work	290 ±20	310±30	830±21	100	100	100	427	100	Deskewed	1D ortho. line profile FWHM from max. voxel	Fused, θ_{OPM} = 35°
E-SPIM, Yang et al. (3)	339 ±18	316±8	596±32	~100	~70	~20	5-7	45	Raw	Not stated	Gaussian illumination beam
			443±29				5-7				Bessel illumination beam
obSTORM, Kim et al. (4)	~400	~500		Not stated	Not stated	100	4 at each depth	175	Raw	Not stated	α = 45° (α is equivalent to OPM angle)
eLife, Sapoznik et al. (5)	299 ±21	336 ±16	731 ±21	180	180	~0	~29	100	Deskewed	3D Gaussian fit	Illumination NA 0.16
Yordanov et al. (6)	269 ±23		657±25	Not stated	Not stated	Not stated	Not stated	100	Raw	Not stated	
Gong et al. (7)	1060 ±100	530 ±50	1750 ±110			100	120	200	Deskewed	1D ortho. line profile FWHM from max. voxel	
Optical tiling OPM, Chen et al. (8)	390 ±50	430 ±40	1220 ±130	800	500	200	943	100	Deskewed	Not stated	
DaXi, Yang et al. (9)	480 ±28	379 ±21	1865 ±174	Stage scan-ning	451	319	156	100	Deskewed then rotated	Gaussian fit to 1D ortho. line profiles	

SCAPE 2.0, Voleti et al. (10)	~500	~1200	~1500			100		200	Deskewed	1D ortho. line profile FWHM from bead centroid	O3 20x/0.6 NA
Lamb et al. (11)	332-576 (Table S2)			25-125 images per stack	150	10			Deskewed	Fourier ring correlation	
SIM OPM, Chen et al. (12)	297 ±20	338 ±15					98	100	Deskewed	Not stated	Non-SIM PSF measurements
Sirinakis et al. (1)	290-315	274-288	712-903	~50 µm radius from optical axis		20	~1000	100	Deskewed	Z plane of best focus and 2D Gaussian fit to determine bead centre. 2D Gaussian fit for XY and 1D Gaussian fit for Z.	
Mantis, Ivanov et al. (2)	Scan 294 ±14	Tilt 289 ±14	Cover-slip 263 ±13	~100	~120	~15	127	100	Raw	3D Gaussian fit or Gaussian fit to 1D ortho. line profiles from bead centre (napari-psf-analysis)	
	294 ±15	262 ±13	683 ±55						Deskewed		
HOPE STORM, Ding et al. (13)	305 ±17	328 ±19	509 ±16	100	100	10		40	Deskewed	Not stated	

Supplementary Table 4: Manufacturer, part number and description of the optical elements in the dOPM system.

Component (see Fig. 1)	Manufacturer	Part Number	Description
Omicron LightHUB	Omicron	LightHUB	Laser combiner with bandpass cleanup filters installed on diode laser sources
		LuxX 445-100	445 nm diode laser
		LuxX 488-100	488 nm diode laser
		LuxX 515-100	515 nm diode laser
		OBIS 561-100	561 nm DPSS laser
		LuxX 642-140	642 nm diode laser
HWP1	Thorlabs	WPHSM05-633	633 nm zero-order half-wave plate
QWP1	Thorlabs	WPQSM05-633	633 nm zero-order quarter-wave plate
HWP2	Thorlabs	WPHSM05-445	445 nm zero-order half-wave plate
QWP2	Thorlabs	WPQSM05-445	445 nm zero-order quarter-wave plate
HWP3	Thorlabs	AHWP10M-580	580 nm achromatic half-wave plate
QWP3	Thorlabs	AQWP10M-580	580 nm achromatic quarter-wave plate
L1	Olympus	UPlan Fluorite 10×	10×/0.3 NA microscope objective
L2	Thorlabs	AC254-150-A-ML	f = 150 mm focal length achromatic doublet
ID (iris)	Thorlabs	SM1D12CZ	Iris diaphragm
BS	Thorlabs	CCM1-BS013/M	50:50 non-polarising beam splitter cube
M1,2,3,4,5,6,8	Thorlabs	PF10-03-P01	Protected silver mirrors
CY1,2	Thorlabs	LJ1629RM-A	f = 150 mm plano-convex cylindrical lens
O5,6	Olympus	UPlan Fluorite 4×	4×/0.13 NA microscope objective
M3,7	Edmund Optics	89623	3 mm protected-silver mirrored prism
O2/3	Olympus	MPLAPON 50x	50×/0.95 NA microscope objective
QWP4	Thorlabs	AQWP10M-580	Achromatic quarter-wave plate
PBS	Edmund Optics	49002	Broadband polarising beam splitter cube, 25 mm
TL2	Ross Optical	L-AOC144/215	Pair of achromatic doublets, adjusted for 1.333× overall magnification of 1st and 2nd microscopes
TL1	Nikon		Internal to Nikon Eclipse Ti2
O1	Nikon	MRD07602	CFI Plan Apochromat VC 60×/1.2 NA WI objective
EM	Semrock	FF01-483/32-25<br>FF01-525/45-25<br>FF01-542/20-25<br>FF01-609/54-25<br>FF01-698/70-25	445 nm (Ex) => FF01-483/32 (Em)<br>488 nm (Ex) => FF01-525/45 (Em)<br>515 nm (Ex) => FF01-542/20 (Em)<br>561 nm (Ex) => FF01-609/54 (Em)<br>642 nm (Ex) => FF01-698/70 (Em)
TL3-1	Thorlabs	TTL100-A	f = 100 mm tube lens
TL3-2	Thorlabs	MVL50M1	f = 50 mm camera lens
sCMOS1,2	Hamamatsu	ORCA-Fusion	Scientific CMOS camera

Supplementary Table 5: Summary of image acquisition parameters for each results figure in the paper. * RF, remote refocus scanning; SS, stage scanning.

Figure	Channels / Dyes	Description	dOPM Mode*	Frame rate / Exposure / Pixels	Ex (nm)	Em Filter (nm)	TL3 (mm)	Pixel Size (µm)	Z step µm / Planes)	dOPM Views
Fig. 1(a-c) & S. Figs. 1-4	100 nm TetraSpeck beads in agarose	Spatial resolution quantification	3D RF	10 Hz / 100 ms / 2048×2048	488	525/45	200	0.086	0.5 µm / 301	2
Fig. 1(d)	Fixed TNBC organoids, labels: FITC-actin, SPY650-DNA	10 organoids, fixed, dual-color	3D RF	5–10 Hz / 100, 50 ms / 2048×2048	488, 642	525/45	200	0.086	1 µm / 151	2
Fig. 2, Movie 1-3	Live TNBC PDOs, labels: collagen, actin, DNA	80 organoids, 48 timepoints, 30 min interval	3D RF	50 Hz / 10 ms / 512×512	488, 561, 642	525/45, 609/54, 697/58	50	0.35	1 µm / 151	2
Fig. 3 & S. Fig 9 & Movie 4-5	Lung slices, labels: ERK FRET, E-cadherin Alexa 647	30 nodules, 144 timepoints, 10 min interval	3D RF	50 Hz / 10 ms / 512×512	445 (x2), 642	483/32, 525/45, 697/58	50	0.35	1 µm / 151	2
Fig. 4	FUCCI spheroids, labels: Cyoplasmic GFP, FUCCI (Venus, mCherry), H2B-mCerulean	~590 spheroids, 4-day imaging, 12 h interval	3D RF	50 Hz / 10 ms / 512×512	445, 488, 515, 561	483/32, 530/43, 560/25, 630/69	50	0.35	1 µm / 151	2
Fig. 5	Fixed single TNBC cells in collagen, labels: actin, tubulin, DNA	~15,000 cells, stage scanning in 96-well plate	3D SS	65 Hz / 10 ms / 512×512	488, 561, 642	525/32, 609/54, 697/58	50	0.35	1 µm / 3000	2
S. Fig. 7 (c&f)	200 nm TetraSpeck beads on coverslip	Collection efficiency quantification	2D	10 Hz / 100 ms / 128×128	488	525/45	375	0.046	NA	flat mirror and both views for 17.5° and 22.5° mirrors
S. Fig. 8	200 nm TetraSpeck bead monolayer	Photobleaching calibration	2D	- / 30 ms / 512×512	488	525/45	50	0.35	- / -	-
S. Fig. 8	Live TNBC organoids, label: mEmerald	Photobleaching comparison, 16 organoids, time-lapse	3D RF	100 Hz / 10 ms / 512×512	488	525/45	50	0.35	1 µm / 151	2

S. Fig. 10 & Movie 6	Fixed GS605 patient-derived tumour organoids	10s of organoids embedded in a dome of basement membrane extract in single well of 96-well plate	3D SS with stitching	63 Hz / 10 ms / 512×512	488, 561, 642	525/32, 609/54, 697/58	50	0.35	1 µm / 1000 per stripe, 5 stripes	2 views, 5 fused stitched volumes
Table 1	Alexa Fluor 488 antibody thin fluorescent sheet	Z-sectioning / light sheet thickness quantification	3D RF	NA / 10 ms / 2048×2048	488	525/45	200	0.88	0.5 / 91	both views for 17.5° and 22.5° mirrors

Supplementary Table 6: parameters used for comparing photobleaching between dOPM and epi-fluorescence imaging.

dOPM		Epi-fluorescence	
Parameter	Value	Parameter	Value
Laser wavelength	488 nm	Excitation filter 1	FF01-488/10-25
		Excitation filter 2	FF01-466/40-25
		Dichroic	FF495-Di03-25x36
Emission filter	525/45 nm	Emission filter	525/45 nm
sCMOS exposure time	10 ms	sCMOS exposure time	10 ms
Laser power Omicron	50% (of 100mW)	LED power setting	100%
Laser power NIS Elements	6% (~ 3 mW)		
Plane spacing (z')	1 μm	Plane spacing (z)	1 μm
No. of planes	151	No. of planes	151
No. of dOPM views	2		
No. of time points	300	No. of time points	300
Pixel size (x', y')	0.35 μm	Pixel size (x, y)	0.108 μm

Supplementary Methods

Fluorescent bead sample preparation

Sparse 2D layers of TetraSpeck™ Microspheres, 0.1 µm (T7279, Thermofisher) or 0.2 µm diameter TetraSpeck™ fluorescent beads (T7279, ThermoFisher) were prepared by diluting the stock solution in Milli-Q (MQ) by typically 500:1. To avoid aggregation, stock solution was agitated for 10 seconds on an Eppendorf vortex prior to dilution. Clean microscope slides were coated in poly-L-lysine by pipetting on 1 mL of 1% poly-L-lysine dissolved in MQ, leaving for 10 minutes and excess 1% poly-L-lysine was washed away by pipetting MQ against the slide surface. Then a volume of 250 µL of bead solution was spread across slide and left for 10 minutes and excess solution was removed by pipetting MQ against the slide surface.

3D volumes of TetraSpeck™ Microspheres, 0.1 µm (T7279, Thermofisher) were prepared by embedding them in agarose gel formed by aqueous agarose (1% agarose) in a glass-bottomed dish (35 mm diameter, #1.5 thickness glass bottom dish, MatTek). Compared to the stock solution of beads, the final solution was diluted by a factor of 40.

Preparation of thin fluorescent sheet

Wells of a glass-bottomed multi-well plate (Greiner Bio-One 655892) were washed with 0.1 mL of 0.1N hydrochloric acid overnight under 300 rpm constant agitation in an orbital shaker at room temperature. This was followed by three washes with purified water (MilliQ) and the plate was then left to dry. The glass was coated by adding 100 µL of poly-L-lysine (P8920 Sigma-Aldrich) diluted 1:20 in MilliQ and incubated at room temperature for 30 minutes followed by five washes with MilliQ water and left to air dry completely for >2 hours. 1 µL of Alexa Fluor 488-conjugated antibody (A21121 goat anti-mouse AF488 Invitrogen, 2 mg/mL) was diluted in 1 mL of phosphate buffered saline (PBS) with 10 µM of dithiothreitol (DTT) and the mixture was vortexed and sonicated for 5 minutes. 0.1 mL was then added to each well and incubated at 4°C overnight. Each well was then washed 3 times with PBS supplemented with 10 µM DTT and imaged in the same solution.

Image acquisition

Volumetric image acquisition was performed using either remote scanning achieved by translation of M3/M7 in the y-direction or by scanning of the sample stage in the y-direction.

For remote scanning, the PIMAG translation stage was configured to accept a voltage command signal in the range -10 to +10 V corresponding to the full scan range of 5 mm. A digital to analogue converter (PCIe-6343, X Series, National Instruments) was used to generate the PIMAG position control voltage and controlled via Nikon's NIS-Elements software configured as a 'Piezo Z' device. A few-mm translation of M3&7 was used to switch between the two views, with smaller translations used to scan the image plane through the sample for each view. The camera was configured to run in 'Normal Area' mode and 'Free Running' mode, and the global exposure transistor-to-transistor logic (TTL) output of the camera was used to trigger the laser exposure.

For stage scanning, the sample motorised stage ($SCAN^{PLUS}$ IM 130 X 85, Marzhäuser) was controlled by a driver unit (Tango 2 Desktop, AUX I/O option, Marzhäuser). It was configured to output a TTL trigger each time the stage travelled a predefined distance in the y-direction, which was set to 1 µm. This TTL output was used as an external input trigger for the acquisition camera. The camera was configured to run in 'Normal Area' mode and 'Free Running' mode, and the global exposure transistor-to-transistor logic (TTL) output of the camera was used to trigger the laser exposure.

The acquisition parameters for each dataset/figure showing imaging results are listed in Supplementary Table 5. All acquisitions were automated using Nikon's NIS-Elements JOBS scripts.

Image deskewing and registration

Image deskewing and registration were performed as described in reference (14). Briefly, the Multi-View Fusion plugin (15) available in ImageJ was used. The plugin was first used to implement the deskewing affine transformation described in equations 1-5 in reference (14). To align the two views, the automatic bead-based co-registration procedure explained in detail in reference (15) was used to co-register all views and spectral channels using the default parameters (multiview_reconstruction v0.11.5, "Fast descriptor-based (rotation invariant)" algorithm, "Register Dataset based on Interest Points", "interest_points=beads", "transformation=Affine", "fix_views=first view"). With the two views co-registered, the same plugin was used to implement interpolation procedures and multi-view fusion and/or multi-view deconvolution procedures as explained in (15, 16). Exemplar co-registration of fused bead data acquired in three spectral channels is shown in Supplementary Figure 12. If required, the Multi-View Fusion plugin provides the capability to provide a greater degree of non-rigid image registration to co-register multiple spectral channels, but this was not implemented here.

The Multi-View Fusion plugin option to extract an experimentally measured PSF was used to obtain a PSF estimate for each view using images acquired of the agarose-embedded fluorescent bead sample described above. Deconvolution was performed using the default settings with 10 iterations. For displaying orthoplanes of processed data, the Multi-View Fusion plugin was also used to rotate and reslice processed volumes from the frame of each view (primed axes in Supplementary Figure 5) into the microscope Cartesian coordinate frame (non-primed axes in Supplementary Figure 5). Finally, the volumes were exported from the plugin as tiff stacks with the image volume resampled into either 0.35^3 μm^3 voxels or 0.175^3 μm^3 voxels.

Fluorescent bead segmentation and quantification

To quantify the spatial resolution across a 100^3 μm^3 sub-volume centred about the zero-remote refocus position, custom-written MATLAB code was used to analyse 3D intensity distributions of fluorescent beads. Candidate bead locations were identified using a difference-of-Gaussians (DoG) filter, with a spatial scale set to correspond to an expected bead diameter of 4 pixels (approximately 224 nm). Binary segmentation was performed on the DoG volume using a threshold of 200 digital numbers (DN), and their centres estimated using the voxel of maximum intensity within each connected region.

For each detected bead, a 3D sub-volume of size 30 × 30 × 80 pixels (21 × 21 × 56 μm^3 in object space) was extracted from the original (non-DoG filtered) data. Background correction was applied by subtracting the 5th percentile of voxel intensities within the sub-volume. Line profiles were extracted along the x, y, and z directions through the peak voxel, and interpolated at 0.001 µm resolution using cubic splines. Full-width-at-half-maximum (FWHM) values were then computed for each axis. Optical sectioning performance was additionally assessed by summing the sub-volume in xy as a function of z, from which a sectioning FWHM (*Zs*) was computed.

Beads were retained only if they were spatially isolated from other beads and produced non-clipped intensity profiles suitable for FWHM estimation. The resulting FWHM values provided localised estimates of the system resolution and were used to compare image quality across resliced, single-view, fused, and deconvolved datasets.

Quantification of light-sheet thickness using a thin fluorescent layer

To characterise the axial thickness of the light sheet across a 100^2 μm^2 field of view (FOV), we analysed the 3D image of a thin fluorescent sheet. Stacks were acquired for both views and resliced and fused to achieve isotropic voxel dimensions (0.086 µm).

A custom MATLAB pipeline was used to calculate the axial full-width-at-half-maximum (FWHM) of the light sheet at each lateral (x, y) position. Each image volume was first background corrected by subtracting the median intensity of a plane ~15 µm axially from the fluorescent sheet signal peak to remove a uniform offset. To improve signal-to-noise and reduce processing time, volumes were then spatially down sampled in the x and y dimensions using cubic interpolation with a binning factor of 4, resulting in an effective lateral sampling of ~0.345 µm.

For each x–y position in the down-sampled stack, a 1D z-intensity profile was extracted. The profile was converted to object-space coordinates using the known voxel size. A cubic spline interpolation with 0.001 µm resolution was applied, and the FWHM was computed as the axial distance between points where the signal dropped to 50% of the peak value. This process generated a 2D FWHM map of the light-sheet thickness across the FOV. The median and interquartile range (IQR) were calculated and the analysis repeated for two light-sheet angles (17.5° and 22.5°).

To estimate the 95% confidence intervals (CIs) on the median FWHM values, we applied a non-parametric bootstrap procedure (1,000 iterations). For each light-sheet condition and metric (FWHM_x, FWHM_y, FWHM_z, FWHM_zsection), bootstrap samples were drawn with replacement from the full set of per-pixel FWHM values. The median was computed for each resample, and the 2.5th and 97.5th percentiles of the resulting distribution defined the CI bounds.

dOPM photobleaching compared to widefield imaging for equal fluorescence signal

A comparison was performed in mEmerald-expressing organoids on the basis of equal detected signal between dOPM and widefield epifluorescence imaging for an in-focus point object. To obtain equal detected signals, we first imaged isolated 200 nm diameter TetraSpeck™ fluorescent beads (T7279, ThermoFisher) on a coverslip using our standard dOPM acquisition parameters (see Supplementary Table 5); a bead was positioned in the centre of the microscope FOV and in the front focal plane of objective O1, and an image was acquired using the dOPM detection path (sCMOS1, EM) as shown in Supplementary Figure 8(a).

The background-subtracted spatially integrated bead signal for dOPM was then taken to be twice the total signal from the single view to account for the fact that dOPM imaging acquires two image stacks per volume (one per view), with both contributing to the total signal. The total integrated signal from both dOPM views was therefore used when comparing to widefield imaging.

We then switched to the widefield configuration (Supplementary Figure 8(b)) by inserting the appropriate filter cube (EX2, D, EM) and flipping the beam path using the switching mirror (SM) and an image was acquired.

To ensure excitation exposure times matched the camera integration times, both light sources (LED and laser) were triggered using the sCMOS global exposure TTL output. Total bead signal was calculated by summing pixel intensities over a square ROI of 5.25×5.25 μm^2, applied to both modalities after accounting for magnification differences. Background signal was estimated from an adjacent ROI of the same size and subtracted. A narrow-band cleanup filter (Semrock FF01-488/10-

25) was used for widefield LED excitation so the LED spectrum was as similar as possible to the dOPM laser excitation spectrum, and the same emission filter (Semrock FF01-525/45-25) was used in both imaging paths to ensure spectral matching in the detection path. This process was repeated for three beads to obtain an average scaling factor between modalities. This scaling parameter was then used to adjust the widefield LED illumination power and sCMOS exposure time (sCMOS2, EM2) until the background-subtracted integrated widefield signal matched the total dOPM signal (sum of both views) of the same bead. This allowed us to determine the LED illumination and exposure combination on the widefield path that would produce the same total in-plane signal as for dOPM.

Triple-negative breast cancer (TNBC) organoids expressing mEmerald (producing fluorescence signal that was observed through most of the cell) were plated in 4 wells of a 24-well plate (Cat. No. 82426, ibidi GmbH), see Supplementary Methods, and four organoids were manually selected per well.

Time-lapse image acquisition was performed over 300 timepoints, see Supplementary Table 6 and Supplementary Methods for details of acquisition and image analysis.

TBNC PDXO organoid line expressing mEmerald

Detailed GCRC1915 patient-derived xenograft organoid (PDXO) culture conditions and experimental methods can be found in Ratcliffe et al. (17) but key points are outlined below. Organoids were authenticated and tested for mycoplasma contamination by the Cell Services Platform at the Francis Crick Institute. Organoids were generated by plating 1×10^4 cells per well 1 -week prior to imaging in a 24-well µ-Plate from ibidi pre-coated with 83 µL Cultrex RGF Basement Membrane Extract, type 2, PathClear®. Media was changed after 3 days.

For endpoint imaging (Figure 1), fixation was performed with 4% PFA for 15 minutes at room temperature and washed three times with PBS. F-actin was labelled with FITC–phalloidin, and DNA was counterstained using SPY650–DNA (Spirochrome). A fixed organoid was imaged in two spectral channels: FITC–actin (488 nm excitation, 525/45 nm emission) and SPY650–DNA (642 nm excitation, 697/58 nm emission). See Supplementary Table 5 for details of other parameters.

Stable organoid lines were generated using the piggyBac transposon system. The mEmerald-expressing vector was generated using PCR-amplified fragments and cloned into EcoRI-HF and HpaI-digested piggyBac transposon vector by Gibson Assembly (NEB). All constructs were sequence-verified before use. A 1:1 mix of mEmerald-expressing piggyBac and pBase plasmids (4 µg total DNA), along with 12 µL Lipofectamine 2000, was incubated for 5 minutes and then added to 0.5×10^5 resuspended cells. This mix was incubated (37 °C, 5% CO_2, humidified atmosphere) in an ultra-low adherent plate (Cat# 10154431, VWR) for 1–4 hours and then replated under the standard conditions described above. After 4 days the media was changed, and organoids were selected and continuously cultured with 5 µg/mL Blasticidin.

For photobleaching experiments (Supplementary Figure 8) culture media was changed to phenol-red-free imaging media. Imaging media consisted of SILAC Advanced DMEM/F-12 Flex supplemented with 17.5 mM D-glucose, 0.699 mM L-arginine hydrochloride, and 0.499 mM L-lysine hydrochloride, along with additional supplements present in the culture media.

Time-lapse imaging of 300 timepoints at 15 s intervals with the two methods was interleaved: time-lapse dOPM imaging was performed for the first organoid, with the next organoid being imaged using widefield imaging. This was repeated until all 16 organoids had been imaged. All selected

organoids were positioned more than 1 mm from the nearest imaged neighbour to avoid the possibility of light exposure from one organoid affecting another within the same well.

The resulting dOPM data were fused and output as 3D stacks with 0.7^3 µm³ voxels. Widefield data were acquired with 0.108 × 0.108 × 1.0 µm³ voxels. For each organoid, every timepoint in the 3D time-lapse was analysed individually. A global intensity threshold was applied to identify signal-positive voxels, and the centroid (centre of mass) of the resulting binary mask was calculated. A fixed-size cuboidal sub-volume, chosen to be completely within the interior all organoids, centred on the centroid was then extracted: 50 × 50 × 50 voxels for dOPM and 300 × 300 × 50 voxels for widefield, corresponding to comparable physical sizes of ROI in each modality. The total signal within each sub-volume was computed after subtraction of a constant camera offset. Signal traces were then normalized to the first timepoint (t = 0) for each organoid to assess relative photobleaching over time.

Live patient-derived triple-negative breast-cancer organoids

Patient-derived organoids were grown from 1 mm core biopsies taken from triple negative breast cancer patients participating in a non-interventional prospective clinical trial (NCT03238144; https://pubs.rsna.org/doi/10.1148/rycan.240138) approved by an independent review board (reference no. 16/LO/1303). Samples were acquired prior to chemotherapy, and all participants provided written informed consent.

Organoids were cultured from single cells to at least 150 µm in diameter for a minimum of 2 weeks in 3 mg/mL final concentration rat tail collagen I (Corning) plated in a glass-bottom 96-well plate (ibidi µ-Plate, square #1.5H glass bottom). To visualize the extracellular matrix (ECM), Cy3 mono-reactive dye (Cytiva) was conjugated to high-concentration collagen on ice using a bicarbonate buffer (pH 9.3), following the labelling and dialysis protocol detailed by Phillips et al. (18). Cy3-labelled collagen was diluted with unlabelled collagen at a 1:43 mixing ratio to preserve biophysical matrix properties prior to embedding. In parallel, one well was prepared using 200 nm TetraSpek multicolor beads (ThermoFisher #T7280) embedded in 1% low-melting agarose as a registration control for dOPM imaging (see Methods for beads-in-agarose preparation). Organoids were stained overnight at manufacturer-recommended dilutions and as per manufacturer instructions, with SPY650-DNA (nuclei) and FastAct-SPY555 (F-actin).

Eighty PDOs were selected manually across 17 wells of a 96-well plate, with 3–5 PDOs chosen per well, using a two-step pre-finding protocol. First, each well was scanned using a 20× air objective (CFI Plan Fluor 20X, Nikon, MRH00205) and the z-position of the coverslip surface was determined using the Nikon Perfect Focus System. Widefield brightfield tile-scan z-stacks were then acquired at 20 µm intervals (11 planes starting from the coverslip) and used to determine the xyz position of organoids. Final xyz coordinates were manually refined using the dOPM 60× water immersion objective under brightfield illumination.

The manually selected 80 organoids were imaged over 48 timepoints at 30-minute intervals using dOPM. Fluorescence excitation and detection were configured as follows: collagen (488 nm excitation, 525/45 nm emission), actin (561 nm excitation, 609/54 nm emission) and DNA (642 nm excitation, 697/58 nm emission).

Ex vivo tumour xenografts in precision-cut lung slices

All cell line engineering and in vivo preparations were performed as described previously (19). Briefly, EGFR-mutant non-small cell lung cancer (NSCLC) cell lines (H1975) were stably engineered to express CFP linked to the nuclear localisation signal (NLS).

The Francis Crick Institute Animal Welfare and Ethical Review Body and UK Home Office authority provided by Project License 0736231 approved all animal model procedures. Procedures described in this study were compliant with relevant ethical regulations regarding animal research. For in vivo experiments, 8-week-old NSG mice were injected intravenously with 1×10^6 FP-expressing tumour cells. After a 4-week tumour development period, mice were euthanized and lungs were inflated with 2% low-melting point agarose via tracheal cannulation. Solidified lungs were dissected and sectioned into 300 µm slices using a vibratome (Leica VTS-1200s). For imaging, slices were placed on a bed of 1% agarose approximately 100 µm thick to ensure stable mounting. Lung slices were cultured in RPMI supplemented with 10% FBS and 1% Penicillin-Streptomycin and imaged within 48 hours of sectioning. Epithelial architecture was visualized using an Alexa Fluor 647-conjugated E-cadherin antibody (BioLegend, Cat. no. 147308) added at 1/500 dilution 2h prior to the start of imaging.

Time-lapse dOPM imaging was performed over 24 hours at 10-minute intervals. Six wells in an ibidi 24-well plate were imaged, with five tumour nodules manually selected per well based on widefield tile scanning prefinding of whole lung slices from a single plane, resulting in 30 nodules imaged in total.

Imaging was performed using 445 nm laser excitation and detection of CFP emission through a 483/32 nm filter. Alexa Fluor 647 was imaged with 642 nm excitation through a 698/70-25 nm emission filter.

Nuclei were segmented from the CFP channel of the 3D time-lapse image stacks using Cellpose (v3.1.1.1, model type cyto3) with GPU acceleration. Prior to segmentation, 3D Gaussian smoothing was applied using scipy.ndimage.gaussian_filter with $\sigma = (0.5, 0.5, 0.5)$ to reduce noise. Segmentation outputs were manually curated in Napari (v0.5.6) by editing the label layer across timepoints, ensuring consistent labels for tracked nuclei. For each tracked cell, voxel-wise label volumes were analyzed using skimage.measure.regionprops to extract per-timepoint centroid position and volume (voxel count). The largest connected component was retained per label per timepoint, and surface meshes were computed using skimage.measure.marching_cubes. Sphericity was calculated as $\phi = (\pi^{1/3})(6V)^{2/3} / A$, where V is voxel volume and A is the mesh-derived surface area. Tracks were visualized using 3D center-of-mass plots and surface meshes over selected timepoints.

Live murine NPE glioblastoma spheroids expressing FUCCI cell cycle reporter

We utilized a genetically transformed GBM stem cell model (NPE) featuring loss of the tumour suppressor genes *Nf1* (N) and *Pten* (P) and expression of the constitutively active, oncogenic EGFR (E) variant *EGFRVIII* as previously described (20). NPE cells transfected with FUCCI2A were a kind gift from Alex Loftus and cultured as per Loftus et al. (21). These cells express the FUCCI cell cycle reporter system (Cdt1-mCherry and Geminin-mVenus) (22) as well as a Histone H2B marker (mCerulean). Vitrogel (The Well Bioscience, IKVAV High Concentration, TWG007), dilution solution (part of the TWG007 kit) and 5x supplements (as described in (21)) were mixed at a ratio of 25:35:15 and cells were added to achieve a concentration of 1.3×10^5 cells/mL. The mixture was mixed thoroughly and, working quickly, 20 µL of the solution was added to the inner 60 wells of a 96-well plate (Revvity, PhenoPlate 96-well, 6055302). Before the Vitrogel matrix set, the plate was briefly pulsed in a centrifuge for 5 seconds at 300 rpm to promote cell adhesion towards the coverslip in a

thin layer of Vitrogel. After the gel had fully set, the wells were supplemented with NPE media as outlined in reference (21), excluding laminin supplementation. Spheroids were cultured for 3 days before drug treatment was applied using a liquid handling system (Tecan D300e) and then imaged using dOPM 24 hours post treatment. The drugs used were Olaparib (Stratech A4154-APE-10mg), Rapamycin (Stratech, A8167-APE-25mg), Camptothecin (Stratech, A2877-APE-250mg), AZD5363 (Stratech, A1387-APE-5mg), Vorinostat (Stratech, A4084-APE-500mg) and Lactacystin (2B Scientific, SIH-327-200UG).

The z position of the top surface of the coverslip in each well was determined using a 20× air objective and the Nikon Perfect Focus System. A GFP epi-fluorescence image was then acquired in each well using a 4× air objective at a 100 µm offset above the top surface of the well bottom. The NIS Elements General Analysis module was used to identify the x-y coordinates of up to 10 spheroids per well. A further GFP epi-fluorescence image was acquired at the x-y coordinates determined in the previous step using the 60× water immersion objective, and the NIS Elements General Analysis module was used again to obtain a more accurate measurement of the x-y position of each spheroid. This resulted in ~590 spheroids locations. A dOPM image was acquired for each FOV in mCerulean (445 nm excitation and 483/32 nm emission), mVenus (515nm excitation and 560/25 nm emission) and mCherry (561 nm excitation and 630/69 nm emission) channels. An additional well containing fluorescent beads (Spherotech URFP-02-02) was imaged using the same settings and used during fusion of the two views.

After acquisition the dOPM data was first manually filtered to remove any volumes that included more than one spheroid per dOPM volume, where a single spheroid was only partially overlapping with the dOPM volume, or where the same spheroid had been added to the list of positions more than once during the secondary 60× prefind.

Nuclear segmentation followed a nonlinear multiscale top-hat enhancement approach (23, 24). 3D Gaussian smoothing was applied at three scales ($\sigma \approx 1.4, 2.8, 11.2$ µm; kernel sizes 2, 4, 8 voxels at 1.4 µm/voxel), producing images U_1, U_2, and U_3. Two normalized differences, $(U_1–U_2)/U_2$ and $(U_2–U_3)/U_3$, were linearly combined (weights $a_1 = 0.7$, $a_2 = 0.3$) to enhance fine-scale structure. Morphological filtering removed holes and thin artifacts; objects <16 voxels (~3.5 µm diameter) were excluded. A 3D watershed separated touching nuclei, and boundary or out-of-spheroid objects were discarded.

Cells-cycle stage was assigned using raw FUCCI mVenus and mCherry cell intensity values (I_{mVenus} and I_{mCherry}) using manually determined thresholds on I_{mVenus} and I_{mCherry} from a scatter plot showing all cells from the plate.

Stage-scanned imaging of fixed MDA-MB-231 cells

MDA-MB-231 cells were obtained from Janine Erler (University of Copenhagen, Denmark), MDA-MB-468 cells from George Poulogiannis (ICR), and SUM159 cells from the laboratory of Rachel Natrajan (ICR). All cell lines were cultured in RPMI 1640 medium (Gibco, Cat# 11835), supplemented with 10% heat-inactivated fetal bovine serum (FBS) and 1% penicillin-streptomycin. Cells were passaged using 0.25% trypsin-EDTA (Gibco, Cat# 25200056), centrifuged at 1000 rpm for 4 minutes, and resuspended in fresh complete medium. Cell numbers were determined using a Countess automated cell counter (Thermo Fisher) with trypan blue exclusion. All cell lines were routinely tested and confirmed to be mycoplasma-free using the e-Myco Plus Mycoplasma PCR Detection Kit (iNtRON Biotechnology).

Cells were harvested by trypsinisation, centrifuged at 1000 rpm for 4 minutes, and resuspended in a neutralised collagen I mixture composed of 2 mg/mL rat tail collagen I (Corning, Cat# 354249), ultrafiltered H_2O, 5× DMEM, and 1 M HEPES (pH 7.5). The pH of the mixture was adjusted to 7.4 before cell embedding. Final cell concentrations in the collagen mix were 4×10^5 cells/mL for MDA-MB-468 cells and 2×10^5 cells/mL for MDA-MB-231 and SUM159 cells.

Fifty microliters of the cell-collagen suspension were dispensed into each well of a pre-chilled 96-well PhenoPlate (Revvity, Cat# 6055308), and immediately placed in an incubator at 37 °C for 1 hour to allow collagen polymerisation. Outer wells were filled with sterile PBS to minimise dehydration and edge effects. Following polymerisation, 50 µL of DMEM were added to the top of each well, and plates were incubated for 24 hours prior to drug treatments.

Cells were treated with either the BRAF inhibitor Vemurafenib (Selleckchem, Cat# S1267) or the MEK1/2 inhibitor Binimetinib (Selleckchem, Cat# S7007) at final concentrations of 2 µM and 5 µM. DMSO (0.05% v/v) was used as a vehicle control. After 5 hours of incubation at 37°C, cells were fixed at room temperature in 4% methanol-free paraformaldehyde (ThermoScientific, Cat# S126728908) for 1 hour.

Cells were washed with PBS and permeabilised with 0.5% NP-40 (Sigma, Cat# I3021) for 30 minutes at 4°C and then blocked with 3% BSA in PBS for 2 hours at room temperature. Microtubules were labelled using a primary anti-α-Tubulin antibody (Bio-Rad, Cat# MCA78G) at 1:1000 dilution for 2 hours at room temperature. After washing (3 × 20 minutes in PBS), cells were incubated with 1:1000 dilution of goat anti-rat Alexa Fluor 568 secondary antibody (Invitrogen, Cat# A-110770) and 1: 5000 dilution of Alexa Fluor 488 Phalloidin (Invitrogen, Cat# A12379) for 2 hours at room temperature.

Following another 3 × 20-minute PBS washes, nuclei were counterstained with SPY650-DNA (Spirochrome AG, Cat# SC501), 1:10000 solution in PBS for 30 minutes and plates were sealed.

The plate was imaged using the dOPM system configured for stage scanning acquisition - see Methods section 'Image acquisition'. Per well, a 3 mm volumetric stripe centred about the middle of each well was acquired. Each volume consisted of 3000 frames with 1 µm stage steps in the y-direction. Imaging was performed at 63 frames per second (10 ms exposure) with a stage speed of 63 µm s^{-1}. For each stripe, 2 dOPM views and three spectral channels were acquired: Alexa Fluor 488, (F-actin, 488 nm excitation, 525/45 nm emission), Alexa Fluor 568 (α-tubulin, 561 nm excitation, 609/54 nm emission) and SPY650-DNA (nuclei, 642 nm excitation, 697/58 nm emission). Acquisition speed was limited by Nikon NIS-Elements software and not camera frame rate, laser power, or sample signal.

Per well, nuclear-channel volumes were cropped at both axial ends of the stage-scanned acquisition to remove the tips of the parallelepiped acquisition volume. Segmentation was performed in MATLAB using single-level Otsu thresholding followed by a watershed on the distance transform, hole filling and 26-connected component labelling. Small objects were excluded (minimum size corresponding to ~2.6 µm diameter at 0.7 µm voxel size).

Per well, binary masks were then analysed in Python. For each object, the 3D centre of mass was computed using scipy.ndimage.center_of_mass. A histogram of z-centre positions was used to identify the dominant coverslip-associated population. Objects displaced >20 µm above this peak were classified as "off-coverslip," consistent with cells invading into the collagen gel.

Stage-scanned imaging of fixed colorectal organoids

Patient tissues were obtained in accordance with the European Network of Research Ethics Committees, following European, national and local laws. Patient-derived GS605-PD07 organoids were modified to express Krt20-iRFP using the CRISPR-Krt20-iRFP fusion protein knock-in and histone-H2B-EGFP and CAAX-mCherry fusion proteins using lentivirus transfection. They were cultured in basement membrane extract (BME) domes (Cultrex BME Type 2, 3536-005-02). For routine cell passaging, organoids embedded in BME were incubated with TrypLE™ Express Enzyme (1X, no phenol red; Life Technologies, 12604039) at 37°C for 10 to 15 minutes. Mechanical dissociation by pipetting was used to obtain a single-cell suspension. TrypLE™ was quenched with Advance DMEM/F12 (Gibco, 12634010) containing 10 mM HEPES Buffer Solution (15630056) and GlutaMAX (Gibco, 35050038), followed by centrifugation and resuspension in 66% cold BME/media. Cells were plated into pre-heated 6-well culture plates.

Organoids were maintained in Advanced DMEM/F12 medium supplemented with 10 mM HEPES, GlutaMAX, B27 supplement without retinoic acid (Gibco, 12587010), 50 ng/mL recombinant human EGF (Peprotech, AF-100-15), 20ng/mL recombinant human bFGF (basic fibroblast growth factor; Gibco, PHG0026), 100 ng/mL recombinant human Noggin (produced in-house), and 1 µM LY2157299. Cultures were monitored bi-monthly for mycoplasma contamination.

For imaging, organoids were seeded in 5 µL BME domes in pre-heated PhenoPlate 96-well plates (Revvity, 6055302) and cultured for 7 days. Organoids were then fixed with 4% PFA and washed with PBS. To support dOPM data dual-view coregistration, TetraSpeck Microspheres (0.2 µm diameter; Thermo Fisher), diluted 20:1 in BME, were included in one well.

dOPM was performed in stage-scanning mode with five stripes. Each stripe consisted of 1000 frames with 1 µm stage steps in the y direction. Imaging was performed at 63 frames per second (10 ms exposure) with a stage speed of 63 µm s^{-1}. Stripes were positioned with a 20 µm overlap in the x direction between adjacent stripes for registration during stitching. For each stripe, three spectral channels were acquired: histone-H2B (H2B)-GFP (nuclear, 488 nm excitation, 525/45 nm emission), RFP-CAAX (membrane, 561 nm excitation, 609/54 nm emission) and iRFP-KRT20 (keratin, 642 nm excitation, 697/58 nm emission). The 5-stripe 2-view 3-channel acquisition lasted approximately 6 minutes and encompassed ~20 organoids. Acquisition speed was limited by Nikon NIS-Elements software and not camera frame rate, laser power, or sample signal.

The reslicing, fusion and tile stitching of dOPM raw data were performed with the BigStitcher plugin. Transformations were initially derived from an equivalent 5 stripe dataset of fluorescent beads in 3D, taken in an adjacent well. The bead-derived transformations were then applied to the organoid dataset, followed by refinement using sample-based interest points within BigStitcher.

Supplementary Movie Captions

Supplementary Movie 1: Time-lapse multi-FOV dOPM imaging of the TNBC PDO organoids shown in Figure 2(a&b). XY-MIPS. Magenta, actin. In (a), yellow, nucleus. In (b), yellow, collagen.

Supplementary Movie 2: XY (top left), ZY (top right) and XZ (bottom left) orthoslices of FOV indicated by blue dashed box in Figure 2(a). Yellow, nucleus; magenta, actin.

Supplementary Movie 3: XY (top left), ZY (top right) and XZ (bottom left) orthoslices of FOV indicated by blue dashed box in Figure 2(b). Yellow, collagen; magenta, actin.

Supplementary Movie 4: Time-lapse 3D renderings of the 10 FOV shown in Figure 3(b). Grey, nucleus; magenta, E-cadherin. Volume shown, $180\times200\times255$ μm^3.

Supplementary Movie 5: Time-lapse 3D rendering of the 3rd FOV from well B4 in Figure 3(b). Grey, nucleus; magenta, E-cadherin; blue, selected cell's nuclear ROI before division; green & blue, progeny of selected cell after division. Volume shown, $180\times200\times255$ μm^3.

Supplementary Movie 6: Animated 3D rendering of data shown in Supplementary Figure 9: stage-scanned dOPM imaging of a fixed GS605 patient-derived tumor organoid. FOV $826\times1157\times113$ μm^3.

References

1. G. Sirinakis, E. S. Allgeyer, D. Nashchekin, D. S. Johnston, User-friendly oblique plane microscopy on a fully functional commercially available microscope base. *Biomed. Opt. Express* **15**, 2358-2376 (2024).
2. I. E. Ivanov *et al.*, Mantis: High-throughput 4D imaging and analysis of the molecular and physical architecture of cells. *PNAS Nexus* **3**, 323 (2024).
3. B. Yang *et al.*, Epi-illumination SPIM for volumetric imaging with high spatial-temporal resolution. *Nat. Methods* **16**, 501-504 (2019).
4. J. Kim *et al.*, Oblique-plane single-molecule localization microscopy for tissues and small intact animals. *Nat. Methods* **16**, 853-857 (2019).
5. E. Sapoznik *et al.*, A versatile oblique plane microscope for large-scale and high-resolution imaging of subcellular dynamics. *eLife* **9**, e57681 (2020).
6. S. Yordanov *et al.*, Single-objective high-resolution confocal light sheet fluorescence microscopy for standard biological sample geometries. *Biomed. Opt. Express* **12**, 3372-3391 (2021).
7. Y. Y. Gong, Y. Q. Tian, C. Baker, A fully water coupled oblique light-sheet microscope. *Sci Rep* **12**, 5940 (2022).
8. B. Y. Chen *et al.*, Increasing the field- of- view in oblique plane microscopy via optical tiling. *Biomed. Opt. Express* **13**, 5616-5627 (2022).
9. B. Yang *et al.*, DaXi-high-resolution, large imaging volume and multi-view single-objective light-sheet microscopy. *Nat. Methods* **19**, 461-469 (2022).
10. V. Voleti *et al.*, Real-time volumetric microscopy of in vivo dynamics and large-scale samples with SCAPE 2.0. *Nat. Methods* **16**, 1054 (2019).
11. J. R. Lamb, E. N. Ward, C. F. Kaminski, Open-source software package for on-the-fly deskewing and live viewing of volumetric lightsheet microscopy data. *Biomed. Opt. Express* **14**, 834-845 (2023).
12. B. Y. Chen *et al.*, Resolution doubling in light-sheet microscopy via oblique plane structured illumination. *Nat. Methods* **19**, 1419-1426 (2022).
13. Z. Ding *et al.*, Three-dimensional super-resolution imaging of whole cells using a high numerical aperture oblique-plane microscope. *Opt. Lett.* **50**, 3529-3531 (2025).
14. H. Sparks *et al.*, Dual-view oblique plane microscopy (dOPM). *Biomed. Opt. Express* **11**, 7204-7220 (2020).
15. S. Preibisch, S. Saalfeld, J. Schindelin, P. Tomancak, Software for bead-based registration of selective plane illumination microscopy data. *Nat. Methods* **7**, 418-419 (2010).
16. S. Preibisch *et al.*, Efficient Bayesian-based multiview deconvolution. *Nat. Methods* **11**, 645-648 (2014).
17. C. D. H. Ratcliffe *et al.*, Multimodal profiling unveils a reversible basal-like breast cancer cell state resistant to AKT inhibition. http://dx.doi.org/10.1101/2025.06.06.658331.
18. T. A. Phillips *et al.*, A method for reproducible high-resolution imaging of 3D cancer cell spheroids. *J. Microsc.* **291**, 30-42 (2023).
19. A. Le Marois *et al.*, Imaging of MAP kinase dynamics reveals endocytic regulation of pulsatile signalling and network re-wiring in response to targeted therapy in EGFR-mutant non-small cell lung cancer. http://dx.doi.org/10.1101/2024.05.14.594112.
20. E. Gangoso *et al.*, Glioblastomas acquire myeloid-affiliated transcriptional programs via epigenetic immunoediting to elicit immune evasion. *Cell* **184**, 2454-2470.e2426 (2021).
21. A. E. P. Loftus *et al.*, An ILK/STAT3 pathway controls glioblastoma stem cell plasticity. *Developmental Cell* **59**, 3197-3212.e3197 (2024).
22. R. L. Mort *et al.*, Fucci2a:A bicistronic cell cycle reporter that allows Cre mediated tissue specific expression in mice. *Cell Cycle* **13**, 2681-2696 (2014).

23. A. F. Santos *et al.*, Angiogenesis: an improved in vitro biological system and automated image-based workflow to aid identification and characterization of angiogenesis and angiogenic modulators. *Assay Drug Dev Technol* **6**, 693-710 (2008).
24. L. Guglielmi *et al.*, Smad4 controls signaling robustness and morphogenesis by differentially contributing to the Nodal and BMP pathways. *Nat Commun* **12**, 6374 (2021).

High Content 3D Imaging by Dual-View Oblique Plane Microscopy

Supplementary Simulation Methods – Description of Vectorial Raytracing Method

Ray generation

Ray angles were generated using the Fibonacci spiral method (see below) to space the rays over2π steradians. Ray angles are defined in polar coordinates by the polar and azimuthal ray angles respectively (θ, ϕ), see Supplementary Simulation Methods (SSM) SSM Figure 1(a).

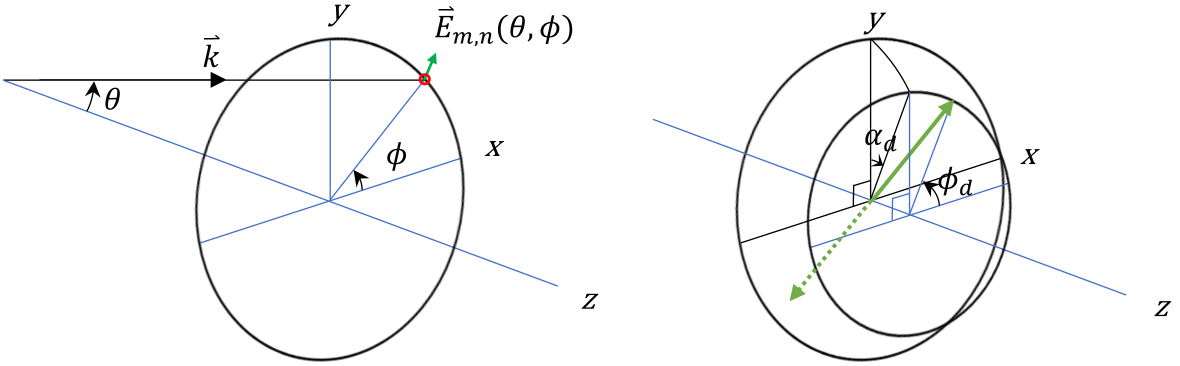

SSM Figure 1 – coordinate systems used to define (a) ray and (b) dipole angles. θ and ϕ are the polar and azimuthal ray angles respectively, while α_d and ϕ_d describe the dipole orientation. (b) depicts dipoles orientations on a unit sphere.

SSM SSM Figure 2 shows an example ray angle distribution. For simulations in this paper, 15,000 rays were generated over 2π steradians (a full hemisphere), and the number collected by O1 depends on its collection half-angle; rays with an angle θ greater than the collection half-angle are rejected.

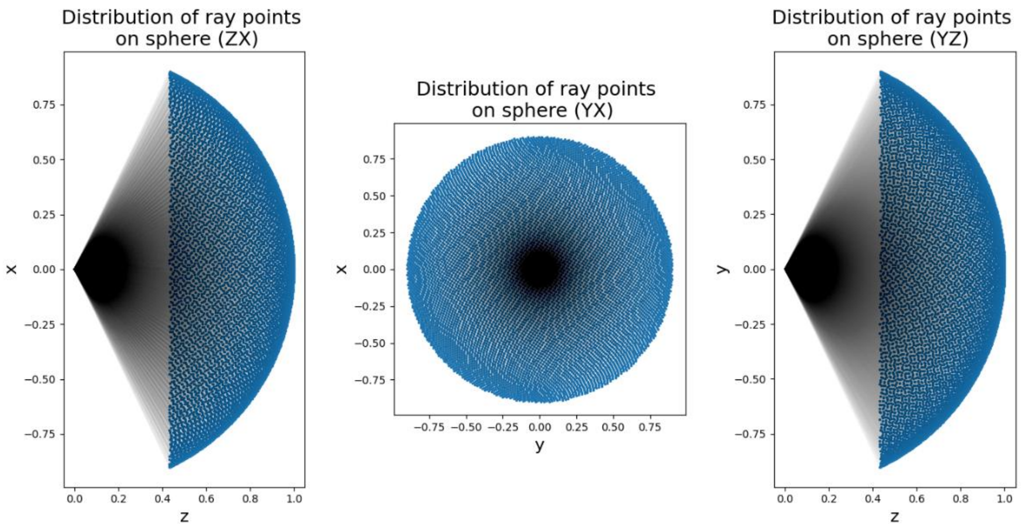

SSM Figure 2 – the cone of generated rays collected by an NA=1.2 water immersion objective, viewed in (left) xz, (centre) xy, and (right) yz planes.

Dipole generation and photoselection

Dipole orientations were simulated in the same manner as for the angular distribution of rays, i.e. using the Fibonacci spiral method, this time with coordinates labeled (α_d, ϕ_d), noting that α_d is defined as being measured from the y-axis — see SSM SSM Figure 1(b). The dipole count for simulations in this paper was 7,500. Photoselection, the preferential excitation of fluorophores based on the excitation dipole orientation relative to the excitation polarization, was modeled classically by scaling the intensity emitted by dipoles by

$$\cos^2\Delta = (\cos\alpha_{ex}\cos\phi_{ex}\cos\alpha_d\cos\phi_d + \cos\alpha_{ex}\sin\phi_{ex}\cos\alpha_d\sin\phi_d + \sin\alpha_{ex}\sin\alpha_d)^2 \quad \text{(SSM 1)}$$

where α_{ex} and ϕ_{ex} are the polar and azimuthal angles of the excitation polarization, which are defined in the same way as the dipole orientation angles. The effect of photoselection for an excitation polarization aligned with the y-axis is shown in SSM SSM Figure 3. Light sheets can be polarized with the electric field in the illumination plane, which we call p-polarised, or out-of-plane (s-polarised), see SSM SSM Figure 4. The static dipole case and rapid tumbling dipole cases are considered—the former case is where the photoselection scaling $\cos^2\Delta$ is applied, while the latter ignores photoselection. The static dipole case assumes that dipole rotation is negligible on the timescale of the fluorescence lifetime, and the rapid tumbling dipole case assumes that the rotational correlation time is negligible compared to the fluorescence lifetime. The excitation and emission dipoles are assumed to be parallel, so the resulting axis of partial polarization is the same as that of the excitation polarization.

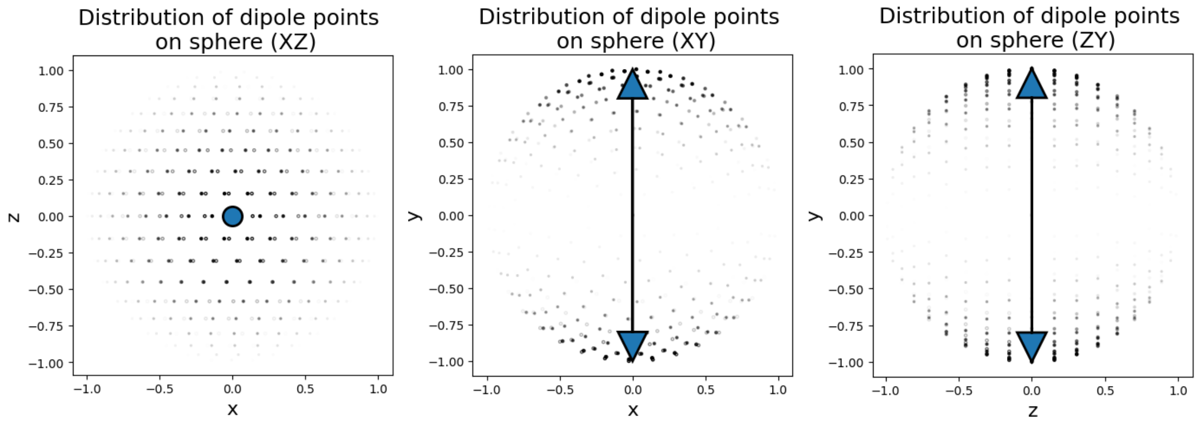

SSM Figure 3 – Example dipole source with 500 dipoles, their orientations indicated by points on a unit sphere. With excitation polarized in the y-axis (blue-tipped arrow), photoselection results in the intensity scaling illustrated by the transparency of the dipole points: dipoles oriented along the z-axis emit with zero intensity, while those oriented along y have maximum intensity. The dipole energy scaling is shown in (left) xz, (centre) xy, and (right) yz plane views.

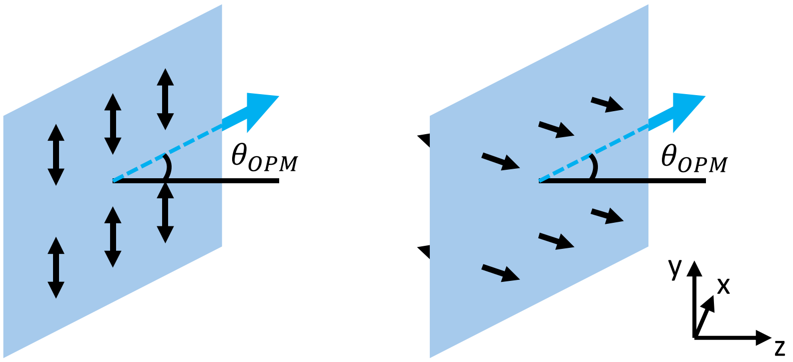

SSM Figure 4 – (left) p- and (right) s-polarized light sheets for an OPM angle of θ_{OPM}, the blue arrow indicating the propagation direction, black arrows the direction of the E-field and the solid black line the optical axis.

<u>Lens operations</u>

Lenses were modelled following the approach implemented by J Kim et al, which considers a lens obeying the Abbe sine condition [1].

The lens is considered solely in the meridional plane for a point object at the front focal point and modelled by a first spherical principal surface with a radius equal to the focal length of the lens and a second, flat principal surface, see SSM Figure 5B. The change of ray direction on intersection of the ray with the first spherical surface requires a change of basis from Cartesian lab coordinates to meridional coordinates. This matrix is given by

$$\mathcal{R}_z(\phi) = \begin{bmatrix} \cos\phi & \sin\phi & 0 \\ -\sin\phi & \cos\phi & 0 \\ 0 & 0 & 1 \end{bmatrix} \qquad \text{(SSM 2)}$$

where ϕ is the azimuthal ray angle, see SSM Figure 4a and 1a.

Following this change of basis, the lens matrix can be applied. The lens matrix maps rays from a spherical principal surface to a second flat principal surface if the lens is collimating the rays (e.g. O1), and vice versa if the incoming rays are collimated (e.g. TL1). The lens matrix is given by

$$\mathcal{L}(\theta) = \begin{bmatrix} \cos\theta_{lens} & 0 & \sin\theta_{lens} \\ 0 & 1 & 0 \\ -\sin\theta_{lens} & 0 & \cos\theta_{lens} \end{bmatrix} \qquad \text{(SSM 3)}$$

where θ_{lens} is the angle of the ray in the meridional plane to the optical axis, see SSM SSM Figure 5.

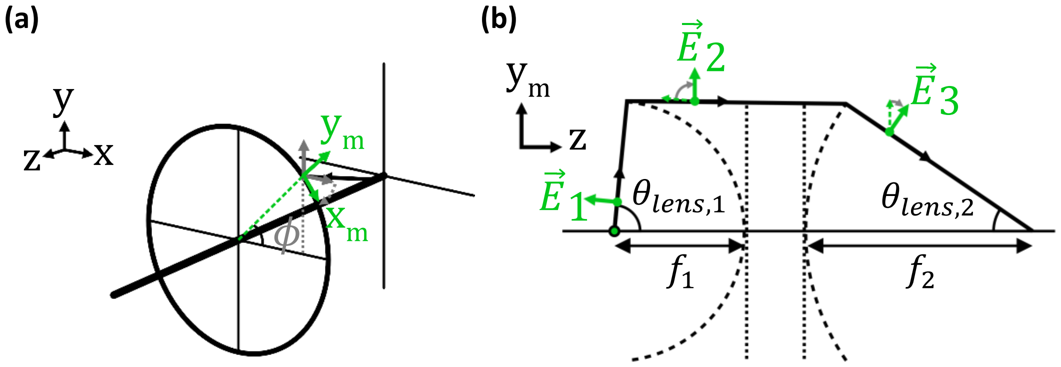

SSM Figure 5 – (a) Illustration of the coordinate system (green) used to describe points in the meridional plane. (b) Two lenses, 1 and 2, both obeying Abbe's sine condition with a point object in the front focal point of the first lens. Rays are traced from the on-axis point source to a spherical surface with radius equal to the focal length of lens 1, f_1. The electric field and wavevector are rotated about x_m by the angle subtended by the ray path and the optical axis (here $\theta_{lens,1}$). The process is reversed for collimated rays being focused to the image point. Here, the ray propagates in the +z direction.

Linear polariser

The linear polarizer transmission axis orientation is described by the azimuthal angle ψ which is defined from *+x* in (and constrained to) the xy plane in lab coordinates, see SSM **Error! Reference source not found.**. The associated optical transfer matrix is given by

$$\mathcal{P}(\phi) = \begin{bmatrix} \cos^2\psi & \sin\psi\cos\psi & 0 \\ \sin\psi\cos\psi & \cos^2\psi & 0 \\ 0 & 0 & 1 \end{bmatrix}. \tag{SSM 4}$$

The linear polarizer is used to model the PBS – the transmission through the PBS as one linear polarizer and reflection as another with polarization axis crossed with the first. This matrix acts in the Cartesian coordinate system and is only used when rays are collimated (see optical setup diagram).

Waveplate

The general Jones matrix for a waveplate is:

$$\mathcal{W}(\delta, \psi) = \begin{bmatrix} \cos(\delta/2) + i\cos(2\psi)\sin(\delta/2) & i\sin(2\psi)\sin(\delta/2) & 0 \\ i\sin(2\psi)\sin(\delta/2) & \cos(\delta/2) - i\cos(2\psi)\sin(\delta/2) & 0 \\ 0 & 0 & 1 \end{bmatrix} \tag{SSM 5}$$

where δ is the retardance or phase difference induced by the waveplate between the polarization components in the fast and slow axes, and ψ is the angle that defines the azimuthal angle of the fast axis from *+x* in the *xy* plane. Quarter waveplates ($\delta = \pi/2$) are used in dOPM.

Mirror

We model the reflection of a ray on a titled planar metallic mirror with a thin protective coating. The tilt θ_{mirror} about the y-axis (vertical on the optical bench) is considered, which describes the normal vector $\widehat{N}$ which is (0,0,-1) (x,y,z) for a tilt of zero degrees—see SSM SSM Figure 6(a). This method allows an arbitrary mirror orientation to be chosen.

Change of basis matrix

The Fresnel matrix operates in the p, s basis, so the basis is changed from the Cartesian lab coordinates. The p and s basis vectors are given by

$$\hat{s} = \hat{k}_{in} \times \hat{N}, \tag{SSM 6}$$

$$\hat{p} = \hat{k}_{in} \times \hat{s} \tag{SSM 7}$$

where $\hat{k}_{in}$ is the incoming wavevector (in Cartesian coordinates) and $\hat{N}$ is the surface normal of the mirror.

The change of basis matrix is then given by

$$M_{ps} = \begin{bmatrix} p_x & p_y & p_z \\ s_x & s_y & s_z \\ k_x & k_y & k_z \end{bmatrix} \tag{SSM 8}$$

where p_i, r_i and k_i are the *i* component of $\hat{p}$, $\hat{s}$ and $\hat{k}$ respectively with $i = \{x, y, z\}$; this is equivalent to combining the dot products of an input vector with each of the basis vectors. These vectors are defined in SSM Figure 6(a).

Thin-film Fresnel reflection matrix

Consulting results from Tompkins [2] for a reflective surface with a thin-film, the complex reflection coefficients for the p and s components is given by

$$\tilde{r}_i = \frac{r_{i,12} + r_{i,23}e^{-i2\beta}}{1 + r_{i,12}r_{i,23}e^{-i2\beta}}, \quad i = \{p, s\} \tag{SSM 9}$$

where $r_{i,12}$ and $r_{i,23}$ are the Fresnel reflections from interfaces between medium 1 and 2 and between 2 and 3 respectively; medium 1 corresponds to air, 2 the thin film material and material 3 is the metal (in this case, silver). This is derived by considering the sum of multiple reflections from within the thin film.

Note that the reflection matrix uses the convention that preserves handedness of the p,s coordinate system, but is not physical (see Section 5 of R. Muller's discussion of ellipsometry conventions [3]). The Householder matrix (see below) models the change in direction of the ray and implements the expected change in handedness.

Fresnel matrix

The complex reflection coefficients are combined into the diagonal matrix to act on the electric field and wavevector in the p, s basis. The Fresnel matrix is therefore

$$\mathcal{F}_{ps} = \begin{bmatrix} \tilde{r}_p & 0 & 0 \\ 0 & \tilde{r}_s & 0 \\ 0 & 0 & 1 \end{bmatrix}. \tag{SSM 10}$$

Householder matrix

The Householder matrix is a generalization of a reflection of a vector and operates in the Cartesian lab basis. This implements a reflection of a ray (and electric field) according to an arbitrary plane with described by the surface normal $\hat{N} = (N_x, N_y, N_z)$ and this is given by

$$\mathcal{H}_{\mathrm{xyz}} = \begin{bmatrix} 1-2N_x^2 & -2N_xN_y & -2N_xN_z \\ -2N_xN_y & 1-2N_y^2 & -2N_yN_z \\ -2N_xN_z & -2N_yN_z & 1-2N_y^2 \end{bmatrix} \quad \text{(SSM 11)}$$

and this is applied after the Fresnel coefficients and in lab coordinates.

Total mirror operator

The final combination of matrices which calculates the electric field after a (Fresnel) reflection from a mirror

$$\mathcal{R}_M = H_{xyz}M_{ps}^{-1}\mathcal{F}_R M_{ps} \quad \text{(SSM 12)}$$

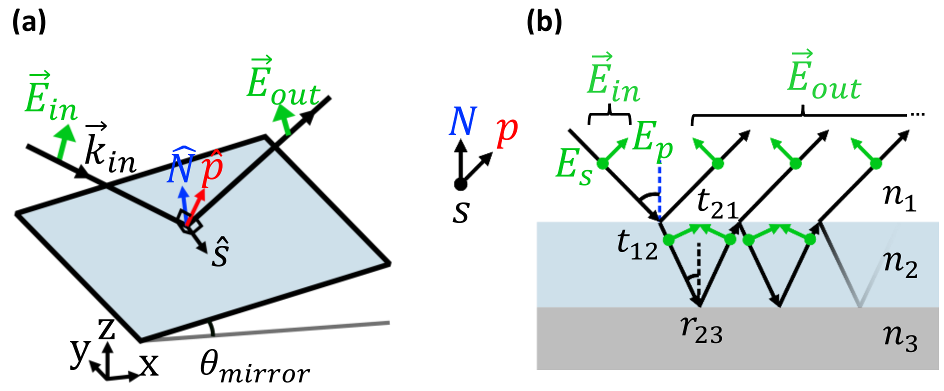

SSM Figure 6 – (a) definition of basis vectors used to describe p- and s-components of electric field. Fresnel reflection coefficients are applied in this p- and s-component basis. *N* is the surface normal, *p* is perpendicular to *s* and $\underline{k}_{in}$, and *s* is perpendicular to *k* and *p*.

Total Jones matrix

The final electric field in the exit pupil of the system is then given by

$$\vec{E}_{m,n}^{out} = M_n^{total}\vec{E}_{m,n}, \quad \text{(SSM 13)}$$

$$\vec{k}_{m,n}^{out} = M_n^{total}\vec{k}_{m,n}, \quad \text{(SSM 14)}$$

where m denotes a specific dipole emitter and n a specific ray, $\vec{E}_{m,n}$ is the initial electric field emitted by the dipole source, $\vec{k}_{m,n}$ is the initial wavevector, and M_n^{total} is the total Jones matrix. For the dOPM system described, this is then given by

$$\boldsymbol{M}_n^{\boldsymbol{total}} = \mathcal{P}\left(\frac{\pi}{2}\right)\mathcal{W}\left(\frac{\pi}{2},\frac{\pi}{4}\right)\mathcal{L}_{O2}\mathcal{R}_z(\phi)\mathcal{H}_{xyz}(\theta_y)\mathrm{M}_{ps}^{-1}\,\mathcal{F}_R\,\mathrm{M}_{ps}\mathcal{R}_z^{-1}(\phi)\mathcal{L}_{O2}\mathcal{R}_z(\phi) \quad \text{(SSM 15)}$$

$$\mathcal{W}\left(\frac{\pi}{2},\frac{\pi}{4}\right)\mathcal{P}(0)\,\mathcal{R}_z^{-1}(\phi)\mathcal{L}_{TL2}(\theta_{lens})\mathcal{L}_{TL1}(\theta_{lens})\mathcal{L}_{O1}(\theta_{lens})\mathcal{R}_z(\phi).$$

Simulated elements in dOPM simulation

SSM Table 1: Elements used in ray-tracing simulation of dOPM system with the Thorlabs protected silver mirror. For SineLens objects, f is the back focal length of the lens.

Type	Element	Parameters			
SineLens	O1	$\mathrm{NA} = 1.2$	$f = 200/60$ mm		$n = 1.33$
SineLens	TL1	$\mathrm{NA} = 1$	$f = 200$ mm		$n = 1$
SineLens	TL2	$\mathrm{NA} = 1$	$f = 162.406015$ mm		$n = 1$
LinearPolariser	LP (PBS transmission)	$\Psi = \pi/2$			
WavePlate	QWP (first pass)	$\delta = \pi/2$	$\Psi = \pi/4$		
SineLens	O2	$\mathrm{NA} = 0.95$	$f = 180/50$ mm		$n = 1$
FlatMirror	Mirror	Film = SiO_2	Substrate = Ag	*d* = 161 nm	Mirror angle = $\theta_{OPM}/2$
SineLens	O2 (second pass)	$\mathrm{NA} = 0.95$	$f = 180/50$ mm		$n = 1$
WavePlate	QWP (second pass)	$\delta = \pi/2$	$\Psi = \pi/4$		
LinearPolariser	LP (PBS reflection)	$\Psi = 0$			

References

[1] J. Kim, Y. Wang, and X. Zhang, "Calculation of vectorial diffraction in optical systems," *Journal of the Optical Society of America A*, vol. 35, no. 4, p. 526, Apr. 2018, doi: 10.1364/JOSAA.35.000526.

[2] H. G. Tompkins and J. N. Hilfiker, *Spectroscopic ellipsometry: practical application to thin film characterization*. Momentum Press, 2015.

[3] R. H. Muller, "Definitions and conventions in ellipsometry," *Surf Sci*, vol. 16, no. C, pp. 14–33, Aug. 1969, doi: 10.1016/0039-6028(69)90003-X.
